# Urine and Fecal ^1^H-NMR Metabolomes Differ Significantly between Pre-Term and Full-Term Born Physically Fit Healthy Adult Males

**DOI:** 10.3390/metabo12060536

**Published:** 2022-06-10

**Authors:** Leon Deutsch, Tadej Debevec, Gregoire P. Millet, Damjan Osredkar, Simona Opara, Robert Šket, Boštjan Murovec, Minca Mramor, Janez Plavec, Blaz Stres

**Affiliations:** 1 Department of Animal Science, Biotechnical Faculty, University of Ljubljana, SI-1000 Ljubljana, Slovenia; leon.deutsch@bf.uni-lj.si (L.D.); simona.konda@gmail.com (S.O.); 2 Faculty of Sports, University of Ljubljana, SI-1000 Ljubljana, Slovenia; tadej.debevec@fsp.uni-lj.si; 3 Department of Automation, Biocybernetics and Robotics, Jožef Stefan Institute, SI-1000 Ljubljana, Slovenia; 4 Institute of Sport Sciences, University of Lausanne, CH-1015 Lausanne, Switzerland; gregoire.millet@unil.ch; 5 Department of Pediatric Neurology, University Children’s Hospital, University Medical Centre Ljubljana, SI-1000 Ljubljana, Slovenia; damjan.osredkar@kclj.si; 6 Faculty of Medicine, University of Ljubljana, SI-1000 Ljubljana, Slovenia; 7 Institute for Special Laboratory Diagnostics, University Children’s Hospital, University Medical Centre Ljubljana, SI-1000 Ljubljana, Slovenia; robert.sket@kclj.si; 8 Faculty of Electrical Engineering, University of Ljubljana, Jamova 2, SI-1000 Ljubljana, Slovenia; bostjan.murovec@fe.uni-lj.si; 9 Department of Infectious Diseases, University Medical Centre Ljubljana, SI-1000 Ljubljana, Slovenia; minca.mramor@kclj.si; 10 National Institute of Chemistry, NMR Center, SI-1000 Ljubljana, Slovenia; janez.plavec@ki.si; 11 Institute of Sanitary Engineering, Faculty of Civil and Geodetic Engineering, University of Ljubljana, SI-1000 Ljubljana, Slovenia

**Keywords:** premature birth, ^1^H-NMR metabolomics, hypoxia, fecal metagenomics, biomarkers, activity, hypoxia

## Abstract

Preterm birth (before 37 weeks gestation) accounts for ~10% of births worldwide and remains one of the leading causes of death in children under 5 years of age. Preterm born adults have been consistently shown to be at an increased risk for chronic disorders including cardiovascular, endocrine/metabolic, respiratory, renal, neurologic, and psychiatric disorders that result in increased death risk. Oxidative stress was shown to be an important risk factor for hypertension, metabolic syndrome and lung disease (reduced pulmonary function, long-term obstructive pulmonary disease, respiratory infections, and sleep disturbances). The aim of this study was to explore the differences between preterm and full-term male participants’ levels of urine and fecal proton nuclear magnetic resonance (^1^H-NMR) metabolomes, during rest and exercise in normoxia and hypoxia and to assess general differences in human gut-microbiomes through metagenomics at the level of taxonomy, diversity, functional genes, enzymatic reactions, metabolic pathways and predicted gut metabolites. Significant differences existed between the two groups based on the analysis of ^1^H-NMR urine and fecal metabolomes and their respective metabolic pathways, enabling the elucidation of a complex set of microbiome related metabolic biomarkers, supporting the idea of distinct host-microbiome interactions between the two groups and enabling the efficient classification of samples; however, this could not be directed to specific taxonomic characteristics.

## 1. Introduction

Preterm birth, defined as a birth before 37 weeks gestation, accounts for approximately 10% of births worldwide. Four degrees of preterm birth are known: extreme preterm (before 28 weeks), very preterm (28–31 weeks), mild preterm (32–33 weeks) and moderate preterm (34–36 weeks) [1]. While the mechanisms underlying preterm birth are complex, with risk factors comprising infection, cervical disease, uterine over-distention, stress and placental disorders [2,3] it remains one of the leading causes of death in children under 5 years of age [4,5]. Improved neonatology care has led to significantly increased pre-term born survival rates over the last 50 years [6]. Importantly, preterm born adults have been consistently shown to be at an increased risk for chronic disorders involving various organ systems, including cardiovascular, endocrine/metabolic, respiratory, renal, neurologic, and psychiatric disorders. These disorders either persist from infancy into adulthood or sometimes even appear in adulthood and result in a moderately (30% to 50%) increased risk of death in early to mid-adulthood in preterm compared to full-term, and even higher risks among those born at the earliest gestational ages [3]. Preterm infants were also shown to experience an imbalance between oxidants and antioxidant capacity [7]. Oxidative stress was shown to be an important risk factor for hypertension, metabolic syndrome (diabetes mellitus, dyslipidemia), lung disease (reduced pulmonary function, long-term obstructive pulmonary disease, respiratory infections, and sleep disturbances) [3].

In addition to the population level disease metrics, preterm born individuals have increased body fat mass, arterial blood pressure, and higher fasting glucose, insulin, and cholesterol levels [3,8,9]. Elevated levels of low-density lipoprotein in preterm individuals pose a greater risk of developing atherosclerosis or cardiovascular disease [10]. Preterm born individuals also experience problems with renal function due to altered nephron development [11]. Imbalance in the ratio between reactive oxygen species (ROS) and antioxidants was identified as conductive to oxidative stress [12], which was associated with increased molecular damage [13]. ROS overproduction was reported to be induced in hypoxia by the xanthine oxidase pathway, catecholamine production and increased rate of electron leakage within the mitochondria [14,15,16,17,18]. Taken together, past research showed that significant differences existed between preterm and full-term born adults with respect to oxidative stress induced by hypoxia, activity, or exercise [7,13,14,19,20].

Preterm birth was also shown to induce life-long pulmonary system effects and compromise ventilator control resulting in blunted hypoxic ventilatory response (HVR) in preterm infants. The PreTerm project (Slovenian Research agency (ARRS) project # J3-7536 (D), Figures S1–S3) was devised to explore whether the differences and impairments in HVR persisted with aging in physically fit young men. The differences in HVR responses between preterm born adults and their age matched full-term controls were explored during rest and exercise, in normoxia and hypoxia [21]. Hypoxia was shown to provoke a similar relative reduction in maximal aerobic power and submaximal ventilatory threshold in healthy preterm and full-term born matched controls with comparable peak oxygen consumption levels. These data suggested that exercising in normobaric hypoxia does not exert a higher ventilator and metabolic load in otherwise healthy physically fit individuals born prematurely [21]. Only recently was the post-exercise accumulation of interstitial lung water shown to be higher in adults born prematurely in hypobaric hypoxia, than in normobaric hypoxia [22].

Given the complexity of the human body and its responses to chronically elevated oxidative stress levels that may persist into adulthood and consequently contribute to the development of numerous noncommunicable diseases observed in the preterm population (diabetes, hypertension or lung disorders) [7], the systemic bodily matrices, such as urine and feces remain surprisingly unexplored by powerful high-throughput top-down analytical approaches [23]. To fill this gap, the aim of this study was to explore differences between preterm and full-term participants’ urine and fecal ^1^H-NMR metabolomes and respective enzymatic reactions, during rest and exercise, in normoxia and hypoxia. In addition, metagenomic analysis of human gut-microbiomes was conducted to assess the differences in the human gut microbiome taxonomy, diversity, functional genes, enzymatic reactions, metabolic pathways and predicted gut metabolites. In this work we hypothesized that significant differences exist between the preterm and full-term groups at the levels of multivariate physiological initial states and their respective responses to the tests conducted (rest or exercise; normoxia or hypoxia) [24].

The analyses of large metabolomic and metagenomic datasets were hypothesized to enable the detection of the characteristic differences between the two groups at information levels not utilized before in preterm research, in addition to the efficient selection of more complex sets of biomarkers by utilizing machine learning and the exploration of vast algorithm spaces.

## 2. Results and Discussion

### 2.1. Group Characteristics in Relation to Gut Physiological Data

Thirty-seven men volunteered for this study and were divided into two groups based on the mode of delivery. Fifteen participants were born at term (full-term control) and 22 were born prematurely (preterm). Control participants were 22 ± 2 years old, weighed 76 ± 6 kg, were 180 ± 5 cm tall, had a VO_2_ max of 52 ± 5 mL kg^−1^ min^−1^, and were born at 39 ± 2 weeks. Preterm participants were 21 ± 2 years old, weighed 69 ± 7 kg, were 175 ± 7 cm tall, had a VO_2_ max of 48 ± 6 mL kg^−1^ min^−1^, and were born at 29 ± 3 weeks. Table S1 shows their baseline data [24]. Gestational age was statistically different between the two groups [24]. Twenty-one preterm and 13 full-term participants were included in the metabolomic and metagenomic part of the PreTerm study. Two full-term and one preterm participant did not collect urine and fecal samples and were excluded from this part of the PreTerm study.

Physiological exercise tests from the preterm project were already published before and showed that incremental cycling in normoxia and hypoxia resulted in increased levels of advanced oxidation protein levels (AOPP), catalase (CAT), superoxide dismutase (SOD), and nitrosative stress markers in both groups (preterm and full-term) immediately after exercise [24]. No differences were observed between normoxic and hypoxic environments. However, hypoxic exposure itself resulted in a significant increase in AOPP, and CAT and showed a trend toward an increase in the nitrosative markers control group only, but not in the preterm group. Further, in line with the above observations, the metabolic response to hypoxia may be blunted in adult preterm born adults [24]. Periodic breathing (repeated oscillations of hyperventilation followed by an apneic phase) was also different in the preterm group than in full-born adults, suggesting a possible physiological mechanism [25]. The hypoxic ventilatory response at rest was lower in preterm, but no differences in exercise were observed between the two groups [21]. Preterm born adults experienced reduced physical capacity in normoxia compared to those full-term born and have a lower hypoxic ventilatory response (HVR, ability to change ventilation in the function of blood oxygen saturation), while no such difference was observed under hypoxic conditions [21,26,27,28,29,30,31]. These reports show that preterm individuals nevertheless exhibited increased oxidative stress, antioxidant activity, and NO metabolism in acute exercise. However, under hypoxic conditions, the preterm group did not exhibit increased levels of plasma advance oxidation protein products (AOPP), catalase, and nitrosative stress markers (NOx) levels, indicating a possibly greater activation of responses resisting oxidative stress under hypoxic conditions [24,32,33].

**Figure 1:**
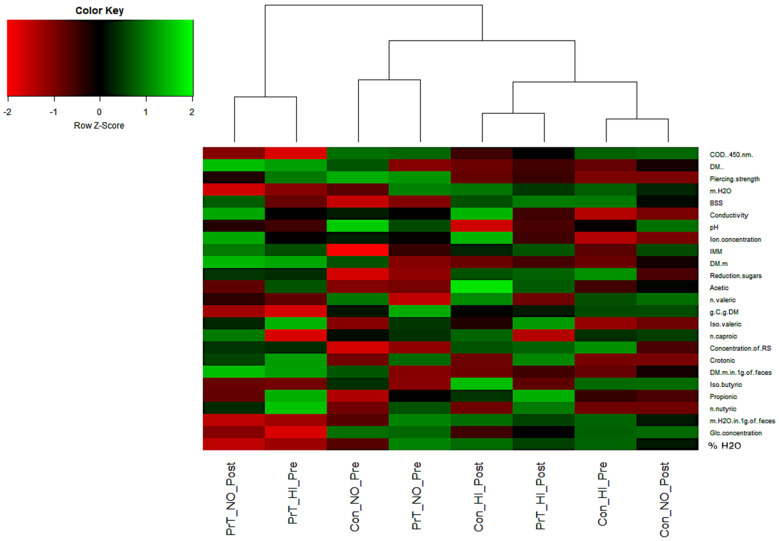
A heatmap summarizing the differences in the 25 measured parameters (COD—chemical oxygen demand, DM—dry matter, m—mass (g), BSS—Bristol stool scale, IMM—molecular mass index, C—carbon, RS—reduction sugars, Glc—glucose) describing the intestinal environment of the preterm and full-term groups exposed to distinct training regimes of the PreTerm project (Figures S1–S3). No significant difference was observed (PERMANOVA; *p* > 0.05; n permutations = 5000).

Based on the integration of past findings obtained utilizing the same cohorts within the PreTerm project, we speculated that measurable differences existed also in the makeup of the intestinal tract characteristics. Various physiological characteristics of the gut environment were previously associated with numerous non-communicable diseases [34]. To assess these differences in the intestinal environment between preterm and full-term control, 25 additional variables were measured in human feces (Figure 1). The obtained results, surprisingly suggest that no significant differences existed in the measured intestinal parameters between the preterm and full-term participants (Permutational analysis of variance (PERMANOVA); *p* > 0.05; n permutations = 5000). In addition, no difference existed between the gut environmental characteristics before and after normoxic and hypoxic test periods (PERMANOVA; *p* < 0.05; n permutations = 5000). The use of a 3-day sampling series enabled us to conclude that the set of 25 measured parameters reported in this study was either insufficient or not measured at an appropriate scale to detect significant differences between the intestinal tracts of the participants from the two groups. As many of the measured parameters were previously effective for detection of differences in the intestinal environment of the participants involved in the three-week bed-rest campaigns of our previous Planetary Habitat Simulation Project (PlanHab) [35,36,37], the results suggest a lack of long-term differences between physically fit preterm and full-term young male participants at the level of the measured intestinal parameters. The role of matching physical fitness between preterm and full-term groups for health maintenance shows that the differences in the status of intestinal tract environments were notably smaller between the active young males irrespective of the preterm birth and oxidative stress markers detected. From this, a different set of additional parameters arise (e.g., zonulin, α1-antitrypsin, eosinophile-derived neurotoxin, bile acid and derivatives, ionic strength, redox potential, mucus characteristics) focusing more intensively on the gut-feces interface and its interaction with the host. This should be used in future studies focusing on immunological, ion-selective and electrochemical characteristics next to spectral and excitation-emission analyses of dissolved organic compounds in the intestinal tract [35,36,37,38,39]. In contrast to our past work utilizing participants exposed to a tightly controlled environment, diet, water intake, circadian rhythm and level of exercise (in the PlanHab project [35,36,37,38]) the PreTerm project interpersonal variability in the same types of variables might have obscured differences in the intestinal parameters associated with individual lifestyle and food preferences in relation to exercise. The significant differences in physiological parameters measured in the PreTerm project were not reproduced in the measurements of the intestinal parameters in the same participants as described above.

### 2.2. Multivariate Relationships in Urinary and Fecal ^1^H-NMR Metabolomes Supported Significant Differences between Preterm and Full-Term Groups

Urine and fecal samples from the preterm and full-term groups were collected on three consecutive days before and three consecutive days after the hypoxic and normoxic tests (Figures S2–S4). In total, each participant was characterized by 12 samples (three daily consecutive samples before and after normoxic and hypoxic tests). Nonparametric analyses utilizing one-way and two-way PERMANOVA on either urine or fecal identified metabolites showed that preterm and full-term groups differed significantly (*p* < 0.001) at both metabolomic levels. In two-way PERMANOVA the training condition (hypoxic vs. normoxic) was marginally significant for urinary metabolomes (*p* = 0.05), but not for fecal metabolomes. This shows that rapid changes in human physiology take place upon the introduction of exercise, while longer bouts of exercise (weeks) would be needed to detect larger differences between physically fit preterm and full-term participants at both levels, similar to the PlanHab project [35,36,37,38]. Differences in the numbers of detected metabolites per group, test and time of sample collection, and the sum of their concentrations in all studied groups were not significantly different (Table S2). In summary, significant differences were identified in the overall makeup of urinary and fecal metabolomes between the preterm and full-term groups.

### 2.3. Urine ^1^H-NMR Metabolomics

**Figure 2:**
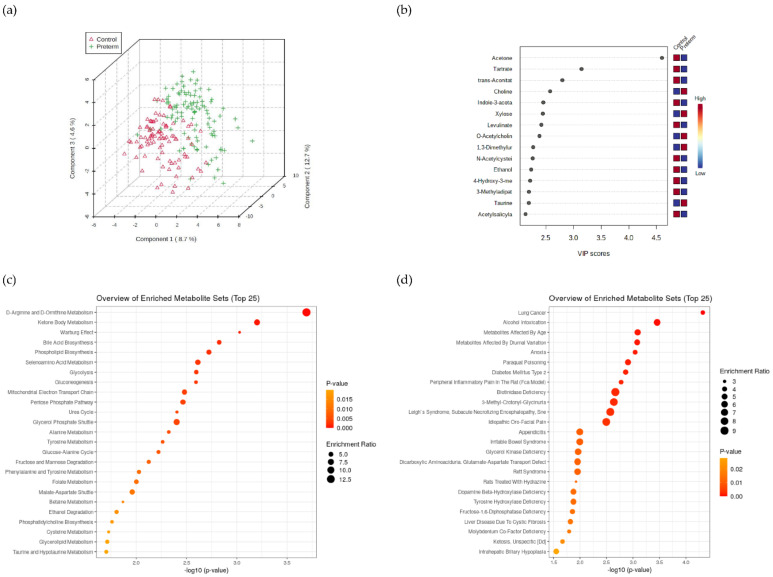
(**a**) Urine metabolomes comparing preterm and full-term born adults and the most differentiating metabolites based on PLSDA method (**b**). The most enriched pathways associated with metabolism (**c**) and diseases (**d**) based on urinary metabolomes. Enlarged (**c**,**d**) figures were added to supplementary (Figures S5 and S6).

The identified differences between the preterm and full-term groups at the level of urinary ^1^H-NMR metabolomes were explored in more detail. When comparing the urine metabolomes (ESM 2) of preterm and control participants using the PLSDA method integrated into MetaboAnalyst [40] and based on cross-validation, three components were recommended to distinguish between the two groups explaining 24% of the variation (Figure 2a,b). Acetone, tartrate, and trans-aconitate [41] were the first three of the most differentiating metabolites in urine, and all three were elevated in the control group based on VIP scores. Acetone metabolism is part of two pathways, the decarboxylation of acetoacetate that is generated during dextrose metabolism and lipolysis, or the dehydrogenation of 2-propanol. Its concentrations in exhaled breath have previously been shown to correlate strongly with acetone concentrations in the blood, as well as with other ketones and were affected by fasting, exercise, and/or disease (e.g.,) diabetes mellitus [42]. Tartrate is part of glyoxylate and dicarboxylate metabolism (Kyoto Encyclopedia of Genes and Genomes (KEGG) pathway: ko00630 (accessed on 20 May 2022) while trans-aconitate (accompanied by creatinine) as the metabolite related to the tricarboxylic acid cycle was indicative of differences in exercise capacity [43].

D-arginine and D-ornithine metabolism, synthesis and degradation of ketone bodies (acetone), and the Warburg effect were the most enriched metabolic pathways (mostly associated with the preterm group) in the urine metabolome (Table S3, Figure 2c,d, Figures S5 and S6). Our results from the metabolome analysis were compared with the metabolomes of specific urinary disease pathways within MetaboAnalyst. Tentatively, the most interesting enriched pathways identified in the preterm group were described before in relation to systemic or tissue hypoxia (Table S4) [44,45].

The MetaboAnalyst PLSDA analysis reported decreased levels of acetone, trans-aconitate and tartrate in the preterm group, irrespective of their matching physical fitness [21,24]. To the best of our knowledge our results could be compared to a single existing study reporting significantly different sets of markers, such as citrate, hippurate, creatinine, and fumarate as crucial metabolites responsible for the differentiation of preterm adults from full-term adults [46]; however, in that study participants of matching fitness and exercise tests were not included.

In the present study, physically fit preterm and full-term participants were enrolled in physical exercise tests and exhibited physical performance indistinguishable between the two groups, i.e., preterm born participants’ physical capacity (expressed as peak oxygen consumption) was not impaired in comparison to normal-born participants [7,21]. In contrast, other studies reported that the physical performance of preterm individuals was lower in normoxia and hypoxia [19] referring to a cohort sampled from the general population. Our study observed a lack of difference in the physical capacity related to the physical fitness of the participants in both groups, further emphasizing the importance of exercise for the maintenance of physical health, while at the same time noticing the differences in metabolic makeup of the two groups at the level of urine. These differences apparently stem from impaired autonomic function as heart rate recovery seems slower in preterm adults and could give rise to anoxia and increase their cardiovascular risk as suggested before [47,48].

### 2.4. ^1^H-NMR Metabolomics of Fecal Content

**Figure 3:**
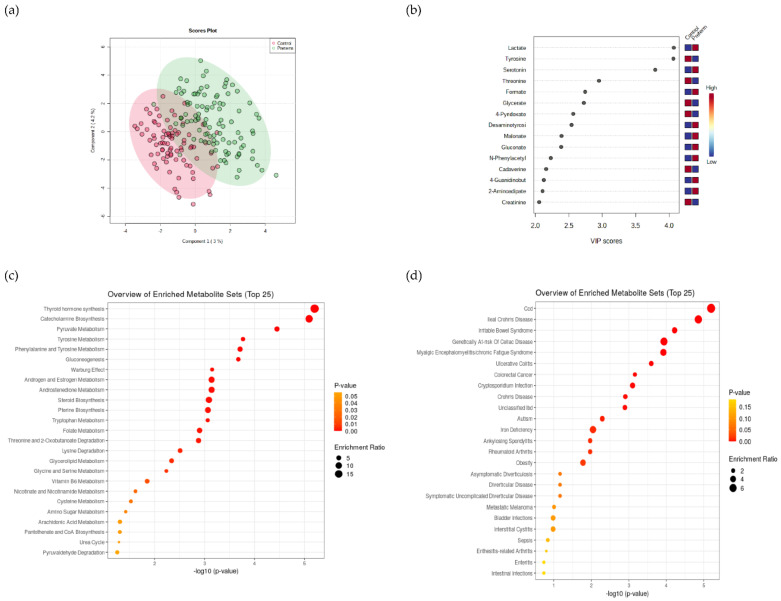
(**a**) Fecal metabolomes comparing preterm and full-term born adults and the most differentiating metabolites based on the PLSDA method (**b**). The most enriched pathways associated with metabolism (**c**) and diseases (**d**) based on fecal metabolomes. Enlarged (**c**,**d**) figures were added to supplementary (Figures S7 and S8).

To match urine sample collection, fecal samples were collected in four 3-day series as described below (Figures S2 and S3, ESM 3). In total, 12 samples were collected per person for the fecal matrix. In contrast to urine metabolomes, two components were sufficient to differentiate preterm and full-term groups in fecal metabolomes by the PLSDA method using MetaboAnalyst [40] (Figure 3a,b). Fecal biomarkers lactate, tyrosine, and serotonin were identified as the three most efficient for differentiation between the two groups. Lactate and serotonin were significantly elevated in the preterm group while tyrosine was decreased. Metabolite set enrichment analysis (MSEA) coupled with the PLSDA reported increased lactate concentrations in the preterm group and reported that pyruvate metabolism and the Warburg effect were enriched in the preterm group. The Warburg effect was also previously associated with mitochondrial dysfunction, which also occurs in preterm infants (Table S5) [49,50]. Thyroid hormone synthesis and catecholamine biosynthesis were the first two most enriched metabolic pathways according to MSEA. In addition, an extended list of fecal metabolites analyzed with MSEA was previously correlated with fecal diseases, such as ileal Crohn’s disease, and irritable bowel disease [51] as the most enriched metabolic pathways (Figure 3c,d, Figures S7 and S8, Table S6).

These results constitute the first report on the significant differences in the metabolomics makeup of fecal samples between the preterm and full-term control groups, irrespective of the observed lack of differences in the 25 measured parameters of the intestinal tract (Figure 1). These results represent possibly the first evidence that systemic differences due to life-long exposure to oxidative stress actually exist and raise the question of whether these differences are linked to minute differences produced from the side of the preterm host or from the side of the microbiome responding to these environmental signals or their mutual interaction in the form of a complex biochemical network steady state.

The rather small extent of variation (7% and 25%) between the two groups could be explained by this approach utilizing feces and urine, respectively, suggesting further and multiple sources of variation exist beyond those described in this study. We extended our interrogation of the data to provide an estimate of the cohort size that would need to be utilized in future experiments. Based on the power analysis module in MetaboAnalyst [40] at least two orders of magnitude larger cohorts amounting to a couple of thousand participants would be required in order to better discern differences at the level of fecal metabolomes. These results point to a conclusion that although fecal metabolomics makeup in physically fit young male participants was significantly different from their matched controls, these differences were independent of the normoxic or hypoxic nature of the tests (PERMANOVA; *p* > 0.05; n permutations = 5000). Apparently, characteristic long-term differences exist between the two groups at the level of fecal metabolomes, most probably linked to the fact that preterm individuals experienced increased oxidative stress, and responded with elevated antioxidant activity, and NO metabolism in the acute exercise studies reported before [24], resulting in the characteristic differences in their fecal metabolomes observed in this study.

### 2.5. Fecal Metagenomics: From Taxonomy, Functional Genes to Predicted Metabolomes

Significant differences in urine and fecal ^1^H-NMR metabolomes between the preterm and full-term groups prompted us to explore whether significant differences exist at the level of the human gut microbiome. Fecal samples collected during the PreTerm project were used for shotgun sequencing. The in-house analytical pipeline utilizing bioBakery [52] was used to preprocess sequence data (Kneaddata (https://huttenhower.sph.harvard.edu/kneaddata/, accessed on 7 April 2022) and analyze the sequences at the strain level of taxonomy (MetaPhlAn3 [53]), diversity (mothur [54]), functional genes, enzymatic reactions and metabolic pathways (HUMAnN3 [53]) next to predicted metabolites (MelonnPan [55]). In total, 853 taxonomic units (kingdoms, phyla, clades, orders, families, genera, and species), 30 diversity calculators, 198,305 gene families, 183,200 enzymatic reactions, 10,974 metabolic pathways, and 80 metabolites present in the human gut microbiota were identified and analyzed. In total, 393,442 variables were considered in this search for differences between the preterm and full-term groups. Each dataset corresponding to a layer of information was analyzed separately using JADBio extensive machine learning modeling as described before [39,56,57].

#### 2.5.1. Taxonomy and Microbial Diversity of Intestinal Tract

In contrast to the observed differences at the urine and fecal metabolomics levels described above, the taxonomic level of information did not result in significant differences between groups (PERMANOVA; *p* > 0.05; n permutations > 5000). In addition, based on the taxonomic data 181,020 JADBio models were trained using an extensive tuning effort, but no reliable biomarker or trained model could be obtained. In general, three different kingdoms were detected in all samples (archaea, bacteria, and DNA viruses). The average relative abundance of bacteria was lower in the preterm than in the full-term group (92.3% vs. 77.5%). The abundance of DNA viruses was higher in the preterm group (7.2% vs. 20%) (Figure S9a). The archaeal kingdom was least prevalent in both groups (0.5% in the control group vs. 2.5% in the preterm group). This lack of significant differences at taxonomic levels was previously attributed to large interpersonal differences between participants [58,59]; however, in this study variance within the full-term control group was at least two times larger than that observed in the preterm group and hence significantly higher (*p* < 0.05) based on the analysis of 3D coordinates after nmMDS and PCoA analysis. This points to the existence of an overarching effect shared by all preterm participants absent from the matching control full-term group.

We further point out that both groups contained matching groups of young healthy physically fit participants, in contrast to past studies exploring the differences between the preterm and general population [60]. Our results point to the fact that some microbiome-related characteristics within the preterm group were apparently shared to a larger extent within the preterm group in comparison to the full-term control group, signifying the existence of differences in the microbial makeup due to differences in the physiology of the host. The existence of an overarching effect shared by all preterm participants absent in the control group was evident from the significantly higher Shannon diversity (*p* < 0.05) (Figure S9b) in the preterm group including other diversity estimates that differed significantly between the two groups (Table S7). These two observations suggest the existence of an interplay between the increased similarity of the major preterm taxonomic categories and the diversity of a smaller highly variable list of taxa not well shared between the preterm participants in this study. It is easily envisioned that additional environmental factors shape the gut microbiome within the preterm group, making the preterm group a narrower subset of the otherwise healthy human gut.

#### 2.5.2. Functional Genes of Human Gut Microbiome

Based on the 198,306 categories describing gene family data, in the preterm and control groups, 90,510 models were trained using an extensive tuning effort in search of biologically meaningful discriminative variables between the preterm and full-term control groups. The entire list of features was used to build and validate a trained model that achieved insignificant validation performance with an AUC and other metrics. Consequently, no significant differences could be identified between the two datasets at the level of functional gene lists.

#### 2.5.3. Enzymatic Reactions Taking Place in Human Gut

**Figure 4:**
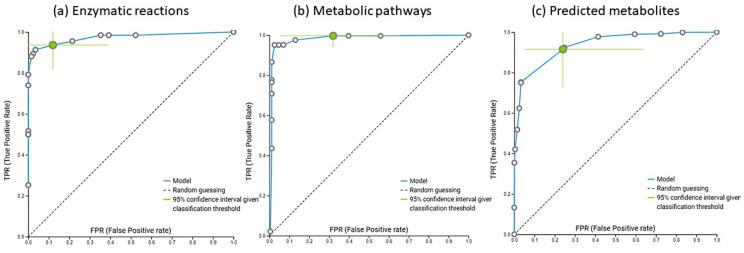
ROC curves of obtained models (JADBio [57]) based on the enzymatic reactions (**a**), metabolic pathways (**b**) and relaxation network predicted metabolites (**c**) produced by our in-house implementation of bioBakery3.

For the aggregation of functional gene information into enzymatic reactions (Figure 4a), again 90,510 models were explored using an extensive tuning effort. The best model was ridge logistic regression with the penalty hyperparameter lambda = 1.0, with an area under the curve (AUC) value of 0.992. In addition to AUC, all other thresholds were also statistically significantly different from the baseline. Features were selected based on the Test-Budgeted Statistically Equivalent Signature (SES) algorithm with the following hyperparameters: maxK = 3, alpha = 0.1, and budget = 3 *n_vars_. RXN-15378, RXN-14971, RXN-21393, and RXN-21394 were equally selected as the most important for discriminating between the preterm and control groups and were increased in the preterm group (Figure S10) (*p* < 0.05). All of the above reactions represent the enzymatic reaction succinate dehydrogenase based on the BioCyc website ([61], accessed on 23 January 2022). We used RXN-15378 to validate the trained model and obtained a validation performance with an AUC of 0.931. Succinate is a metabolite produced by both host and microbial cells and accumulates under conditions of inflammation and microbial imbalances in the intestinal tract [62]. Succinate was shown to accumulate in areas of inflammation and metabolic stress [63] and can have tissue specific but also systemic effects as a proinflammatory signaling molecule [62,64,65,66]. Although gut microbes represent the predominant source of succinate, it is typically rapidly consumed in the production of propionate, one of the major short chain fatty acids, by *Bacteroides* spp., *Prevotella* spp. and some members of Firmicutes [62,67]. Although the mucosal uptake of succinate as a charged molecule over the mucosal epithelia is significantly higher in the small intestine, it takes place to various extents throughout the length of the intestinal tract and requires sodium dependent transport proteins [62]. In addition to the internalized succinate provided by the microbiome, succinate also accumulates within cells under conditions of low oxygen as a metabolic signature of hypoxia, generating HIF-1α to regulate cellular responses and adapt to a low oxygen environment. At normoxia, HIF-1α is regulated by posttranslational hydroxylation and degradation by prolyl-hydroxylase activity that converts alpha-keto-glutarate to CO_2_ and succinate while inactivating HIF-1α. Excess uptake of microbiome produced succinate results in higher levels of intracellular succinate that can slow down prolyl-hydroxylase activity through product inhibition and result in an additional activation and stabilization of HIF-1α beyond its response to hypoxia itself, which can significantly augment the LPS-induced expression of proinflammatory cytokines [62,68].

#### 2.5.4. Metabolic Pathways Observed in Human Gut

Based on the metabolic pathway data (Figure 4b), 60,310 models were trained, with extensive tuning effort. The best model was ridge logistic regression with a penalty hyperparameter of 100 and an area under the curve (AUC) of 0.981. In addition to AUC, all other thresholds were also statistically significantly different from baseline. Features were selected on the basis of Lasso feature selection with a penalty = 1.5. On the basis of the MetaCyc website ([69,70,71]; accessed on 23 January 2022), the most important metabolic pathways were PWY-7456 (β-(1,4)-mannan degradation), PWY-7323 (superpathway of GDP-mannose-derived O-antigen building blocks biosynthesis), GLYCOLYSIS-TCA-GLYOX-BYPASS (a superpathway of glycolysis, pyruvate dehydrogenase, TCA, and glyoxylate bypass), P221-PWY (octane oxidation), and PWY-5173 (unclassified). These pathways were the most important for distinguishing the preterm group from the control group and were all increased in the preterm group (Figure S11). The entire set of selected features was used to validate the trained model and achieved a validation performance with an AUC of 1.00. The relative frequency of this response was significantly increased in the preterm group, which was also confirmed by the t-statistic (*p* < 0.05). A set of selected features was used to validate the trained model and achieved validation performance with an AUC of 1.00.

The β-(1,4)-mannan degradation (PWY-7456) belongs to *Bacteroides fragilis* in the human intestinal tract and is essential for mucosal integrity and host nutrition [72,73]. Degradation of mannan by either *Bacteroides dorei* or *Fecalibacterium prausnitzii* and *Roseburia intestinalis* promotes the growth of *Lactobacillus helveticus* and *Bifidobacterium adolescentis*, which have probiotic properties and promote the synthesis of short chain fatty acids [74] or promote the growth of commensal microbes [75,76].

The superpathway of GDP-mannose-derived O-antigen building blocks biosynthesis (PWY-7323) is involved in lipopolysaccharide (LPS) biosynthesis. Only gram-negative bacteria have LPS, and O-antigen is the part that extends the polysaccharide away from the cell surface and triggers the host cell immune response [77,78]. Gram-negative bacteria observed in preterm infants cause serious infections, such as sepsis [79] coupled with the absence of MD -2 (a protein responsible for the recognition of LPS), which leads to a higher risk of developing intestinal diseases in adults born preterm due to the impaired recognition of LPS in the past [78]. Elevated LPS levels may also contribute to inflammaging (chronic, low-grade inflammation that develops with age) [80]. This also fits our observation that microbially produced succinate coupled with hypoxia can significantly augment LPS-induced expression of the proinflammatory cytokines [68].

GLYCOLYSIS-TCA-GLYOX-BYPASS (a superpathway of glycolysis, pyruvate dehydrogenase, TCA, and glyoxylate bypass), is a superpathway that was significantly overrepresented in the preterm group. It integrates some of the fundamental components of energy metabolism, starting with a hexose sugar and ending with CO_2_ and several forms of highly reducing metabolites that can be used for adenosine triphosphate (ATP) generation. Even though acetyl-CoA is shown in this superpathway as a product of the glycolysis pathway, it is also generated by the degradation of fats and proteins and by the fermentation of many metabolites. This superpathway includes the glyoxylate cycle, which bypasses those steps in the TCA cycle that lead to a loss of CO_2_, and operates in bacteria. The increased energy production in the preterm microbiome apparently coincided with the general characteristics of the preterm individuals, such as increased oxidative stress, elevated antioxidant activity, and NO metabolism in acute exercise as described above [7]. It is possible to suggest that the intestinal conditions experienced by the gut microbiome exerted additional stress on the microbial functioning as well. Further research is needed to corroborate this notion.

In line with our observation of differences in microbiome functioning, P221-PWY (octane oxidation) was shown to increase with the Westernization of the human gut and lifestyle [81,82] as volatile organic compounds, including octane, were found either in the exhaled air or feces of human subjects with diverse medical conditions associated with oxidative stress and chronic inflammation, including lung cancer [82,83] obstructive sleep apnea [84], gastrointestinal diseases [85] and NAFLD [86]. Previous studies demonstrated that several alkane-degrading bacteria were capable of using diverse compounds as a carbon source in addition to alkanes [87], which are further oxidized to fatty acids via the bacterial β-oxidation pathway (BioCyc ID: P221-PWY). The key process in octane oxidation is the alkane hydroxylase system that introduces molecular oxygen in the C1 atom of the hydrocarbons at the expense of NADH to yield primary alcohols [88] that were further linked to liver associated diseases.

The acetyl-CoA biosynthesis (PWY-5173) pathway involved in carbohydrate metabolism was also significantly increased in the preterm group. The resulting acetyl-CoA acts as a precursor in the synthesis of intestinal short chain fatty acids including butyrate and acetate [89], that are important in maintaining gut health [90]. The increased levels of acetyl-CoA biosynthesis fit nicely with the other pathways observed in this study that either contribute or consume mass flow related to this reaction. As both preterm and full-term groups were composed of healthy young physically fit males differing significantly in acetyl-CoA biosynthesis, our findings support a recent report on this pathway being one of the most variable pathways in a survey of subgroups of elite Irish athletes [91].

These overall results of the metabolic pathway analysis point to the fact that (irrespective of the heterogeneous makeup of the underlying microbiome taxonomy within the individual participant) the complex coordinated adjustments to the metabolism of the microbiome nevertheless take place and can be robustly reproduced from the integration of the sequencing information as described in this study [91,92,93,94] and can be linked to physiologically meaningful differences between groups reported before [24].

#### 2.5.5. Predicted Water- and Lipid-Soluble Intestinal Metabolites

Our last layer of information dealt with the extended analysis of sequencing data towards water- and lipid- soluble predicted metabolites utilizing relaxation-network analysis, which has been extensively trained and validated before [52,55]. In summary, 17 metabolites (out of 81) (Table S8) predicted with MelonnPan were detected also by ^1^H-NMR in fecal samples, showing possible interaction between two systemic metabolisms (human and microbial). None of these metabolites were chosen by machine learning. Metabolites associated with the human gut microbiota (Figure 4c) were explored utilizing JADBio and 181,020 models were trained using extensive tuning efforts. The best model was a Support Vector Machine type C-SVC with a radial basis function kernel and hyper-parameter (cost = 10, gamma = 1.0), with an area under the curve (AUC) value of 0.976. In addition to AUC, all other thresholds were also statistically significantly different from the baseline. Feature selection was based on LASSO feature selection with a penalty = 0.25. Alpha-muricholate, putrescine, dimethyllysine, diacetylspermine, and C16 carnitine were significantly increased in the preterm group. In contrast, hydrocinnamic acid, fructose, glucose and galactose, chenodeoxycholate and deoxycholate were lower in the preterm group (Figure S12). When the trained model was applied to the test portion (30% of our total data set) validation performance with an AUC of 0.957 was obtained. In the following sections let us first review the predicted metabolites significantly increased in the preterm group.

Carnitine was increased in the preterm group and is associated with trimethylamine N-oxide (TMAO) production (Figure S12). TMAO is synthesized by the microbiota from trimethylamine (TMA), which in turn is formed from carnitine or choline. Increased choline content in the preterm group was also observed in urine metabolomics. These two molecules together (carnitine and choline), in conjunction with the microbiota, may be the most important cause of the increased likelihood of cardiovascular disease in the preterm group [8,95,96,97,98,99,100].

Putrescine and diacetylspermine are polyamines and important metabolites for the gut microbiota (Figure S12). Putrescine is synthesized by interspecies cooperation between *Escherichia coli* and *Enterococcus faeacalis* and is formed from arginine [101,102]. Elevated putrescine levels have been associated with an older gut microbiota [103], increased gut permeability, and elevated levels of inflammatory cytokines in mouse colon tissue [104]. Elevated putrescine levels led to activation of genes that regulate oxidative stress, which may lead to a parallel increased risk of developing metabolic syndrome [35,36,37,38] and irritable bowel syndrome [105].

Diacetylspermine as a polyamine metabolite was linked to cancer growth and its association with microbial biofilm formation. It is synthesized by bacterial acetylation and has been significantly upregulated in tissues with biofilms in animal models [106]. This suggests that microbial organization and biofilm formation capacity at the interface between the mucus layer and lumen might differ significantly between the preterm and full-term participants, an observation worth further exploration.

Dimethyllysine can be the end product of either host or microbial metabolism but currently little is known about its physiological roles for the host and microbiome in the Human Metabolome Database [107], ChemSpider (https://www.chemspider.com/ (accessed on 15 April 2022)) or FooDB (www.foodb.ca (accessed on 15 April 2022)) (Figure S12). A recent review of macronutrient metabolism by the human gut microbiome focusing on major fermentation byproducts and their impact on host health [108] reported that the major products of lysin were acetate, butyrate and cadaverine, hence linking this compound to the short- and long-chain fatty acid cycles associated with ulcerative colitis [109].

Alpha-muricholic acid was identified by the MelonnPan [55] relaxation network since its first use in the analyses of human samples analyzed using MelonnPan [110,111], suggesting a misclassification of rodent muricholic acid for cholic acid in humans in this approach. Nevertheless, irrespective of its MelonnPan supported assignment, it is evident that this secondary bile acid was identified at elevated levels in the preterm group, fitting into the framework of the distinct chemical makeup of the preterm gut in relation to fat metabolism and the metabolites reported in this study.

In addition to elevated metabolites identified by MelonnPan in the preterm group, the following metabolites were identified in significantly lower concentrations in the preterm group.

Deoxycholate (decreased in the preterm group) is another metabolite that interacts with microbes (Figure S12). Deoxycholate is a secondary bile acid. The human intestinal microbiota (*Bacteroides intestinalis*, *Bacteroides fragilis*, *Escherichia coli*) are involved in the production of secondary bile acids from primary bile acids, such as choline. Deoxycholate is also known to promote colon cancer. Because of the increased cholate levels in the preterm group, we would expect a greater likelihood of microbial metabolites associated with primary bile acids, as well as increased levels of the expected metabolites in the preterm group. In contrast, deoxycholate levels were decreased in the preterm group. This could also be due to the greater urinary excretion of cholate (cholate was increased in the urine of preterm infants). Bile acids also generally induce mitochondria ROS production. Preterm infants are challenged by ROS in the first few months of life, possibly implying that the systemic response is to increased urinary excretion of bile acids in preterm infants [112,113,114,115].

Hydrocinnamic acids are a major class of phenolic acids from dietary fiber with the characteristic phenylpropanoid C6-C3 backbone that were significantly decreased in the preterm group (Figure S12). Although the polyphenol–gut microbiota interactions and their impact on human health have been known for decades, there is great inter-individual variation caused by the different individual capabilities of processing, absorbing and using these compounds effectively [116]. In light of the physiological differences between the two groups analyzed in this study, it seems plausible that differences exist also in the extent of the utilization of these polyphenols in the preterm group. In addition, lower levels of hydrocinnamic acid were observed in patients with Crohn’s disease and ulcerative colitis compared to the healthy cohort. Lower levels of hydrocinnamic acid in the preterm group may lead to increased levels of circulating BCAAs, which in turn predisposes preterm born individuals to metabolic syndrome and cardiovascular disease [117,118,119,120].

The lower levels of reducing sugars fructose, glucose and galactose, in the preterm group corresponded with a greater capacity to produce short chain fatty acids (Figure S12). The metabolic reactions and predicted metabolites jointly suggest the existence of a larger metabolic flow-through of the preterm microbiome in comparison to the full-term group, pointing to significant differences in the environmental setup in the preterm gut.

In conclusion, the results presented here constitute the first report on the differences in the urine and fecal metabolomes between preterm and full-term groups of physically fit healthy young males. Clear differences were identified in the urine and fecal metabolomes next to the metabolic pathways, suggesting that systemic differences between the two groups affect the metabolism of the host as well as intestinal tract parameters and that of the underlying microbiome (Figure S13). One has to realize that studies with female participants are lacking and not many studies with sufficient statistical power were reported so far to close the gap. With the concomitant methodological developments presented in this study, the exploration of the more complex female metabolome and responses to inactivity and hypoxia can be commenced in a comparable way [35].

## 3. Materials and Methods

### 3.1. PreTerm Project: Cardio-Respiratory Responses during Hypoxic Exercise in Individuals Born Prematurely

The PreTerm project aimed to investigate the acute cardio-respiratory responses during rest and exercise in two groups of prematurely born, but otherwise healthy male adolescents and adults. In addition, this project aimed to elucidate the underlying mechanisms of the altered resting and exercise cardio-respiratory responses in prematurely born, but otherwise healthy individuals. The results from this cohort were compared to the data from control groups consisting of healthy, age and aerobic capacity-matched individuals born at full-term resulting in a unique dataset. The obtained results provide extensive basic physiological data on the development of cardiorespiratory control in individuals born prematurely, hypoxia exercise capacity and cardiorespiratory demand during hypoxic exercise in non-acclimatized individuals born prematurely [24].

Thirty-seven healthy men volunteered and gave written informed consent to participate in this study (Cardio-respiratory responses during hypoxic exercise in individuals born prematurely—ARRS research project J3-7536). All participants were free of cardiorespiratory and hematologic disease and had not been exposed to altitudes above 1500 m during the one-month period prior to the study. Twenty-two participants were born premature (gestational age ≤ 32 weeks; gestational weight ≤ 1500 g) and 15 were born full-term. The experimental protocol was approved by the National Medical Ethics Committee of Slovenia (No. 0120-101/2016-2) and conducted in accordance with the principles of the Declaration of Helsinki. The study was also pre-registered at ClinicalTrials.gov (NCT02780908) [24].

The experimental protocol included two testing sessions in each group. On both occasions, no more than seven days apart, participants performed a graded exercise test for voluntary exhaustion. During the exercise tests, participants breathed either normoxic ambient air (fraction of inspired oxygen (FiO_2_ = 0.209) or a humidified hypoxic air mixture (FiO_2_ = 0.130 corresponding to a terrestrial altitude of approximately 3800 m) in a randomized, placebo-controlled manner. Indirect calorimetry, near-infrared spectroscopy and ECG measurements were performed during all tests. During both tests, participants performed a hypoxia sensitivity test to assess the hypoxic ventilatory response at rest and during exercise. In addition, selected hematological and oxidative stress markers were determined from blood samples collected before and after each hypoxia sensitivity test [24].

The two graded exercise tests were performed on an electromagnetically braked cycle ergometer (Ergo Bike Premium, Daum electronics, Fürth, Germany) under normoxic (FiO_2_ = 0.21; PiO_2_ = 147 mmHg) and normobaric hypoxic (FiO_2_ = 0.13; PiO_2_ = 91 mmHg) conditions in a randomized manner. They were blinded as to the FiO_2_ of the gas mixture they inhaled on both occasions. Both tests were performed at the same time of day for each participant. The test protocol started at 60 W and was increased by 40 W every 2 min until exhaustion. The normoxic and hypoxic tests were performed exactly 7 days apart. During the tests, participants breathed through a face mask (Vmask, 7500 series, Hans Rudolph Inc., Shawnee, KS, USA) and oxygen uptake (VO_2_) and ventilation (VE) were measured using a metabolic cart (Quark CPET, Cosmed, Rome, Italy). Capillary oxygen saturation (SpO_2_) was measured using a transcutaneous finger pulse oximetry device (Nellcor, BCI 3301, Boulder, CO, USA). Fecal and urine samples were collected three consecutive days before and three consecutive days after the test under normoxic and hypoxic conditions (Figure S1) [24].

### 3.2. Sample Collection

Fecal and urine samples were collected three consecutive days before and three consecutive days after the test under normoxic and hypoxic conditions at the home of the participants (Figures S2 and S3). Collected samples were frozen at −20 °C immediately after collection. All participants collected 12 urine and 12 fecal samples in total. Three urine and three fecal samples were collected before and after normoxic tests, giving rise to six urine and six fecal samples per participant. The same approach was utilized for hypoxic tests, giving rise to another six urine and six fecal samples per participant. Two full-term and one preterm participant did not collect fecal and urine samples before and after the exercise test and were excluded from the metagenomic and metabolomic part of the PreTerm study.

### 3.3. ^1^H-NMR Metabolomics

Samples were thawed at room temperature before preparation for NMR measurements. All collected samples were centrifuged (1.5 mL) at 10,000× *g* for 30 min to remove fine particles. Subsequently, 400 µL of the supernatant was mixed with 200 µL of ^1^H-NMR buffer as previously described [121] and stored at −25 °C until analysis.

Prior to analysis, samples were thawed at room temperature and transferred to a 5 mm NMR tube. TSP was used as an internal standard for quantification, as described previously [121].

A 600 MHz Bruker Neo NMR spectrometer equipped with a 5 mm HCN Cold probe was used to record NMR spectra at 25 °C. The ^1^H NMR spectra of the samples were recorded with a spectral width of 9.0 kHz, a relaxation delay of 2.0 s, 32 scans and 32 K data points. A double pulsed field gradient spin echo (DPFGSE) pulse sequence was used to suppress water. The total correlated spectrum (TOCSY) was measured with 1H spectral widths of 7.0 kHz, 4096 complex points, a relaxation delay of 1.5 s, 32 transients, and 144 time increments. An exponential function and a cosine squared function were used for apodization. Zeros were filled before the Fourier transform. TopSpin (version 4.1) was used to process the NMR spectra [37,38,39,56,122].

#### Spectra Processing

NMR spectra were preprocessed with an internal script and prepared for identification with the Chenomx Compound Library, extended to the Human Metabolome Database [41,107], giving access to the chemical shift profiles of 674 compounds used in the analyses. Chemical shifts of 647 compounds were used for the identification of metabolites observed in our study. The resulting spectra were then analyzed with targeted quantitative metabolomics using Chenomx NMR Suite version 8.6 (Chenomx, Inc., Edmonton, AB, Canada). ChenomX profiler was used for randomized spectral fitting. All spectra were processed in the same way by spectral deconvolution and once metabolites were identified, urine and fecal data matrices were established, assigning 0 to a particular metabolite not detected in all samples.

### 3.4. Fecal Metagenomics

Fecal samples collected three days before and 1 day after normoxic and hypoxic testing were used for shotgun sequencing; 200 mg of feces were used for DNA extraction using the MagicPure Stool and Soil Genomic DNA Kit (Beijing, China) according to the manufacturer’s protocol. Shotgun sequencing was performed using TruSeq Nano DNA (350) (Macrogen, Seoul, Korea).

#### Sequence Processing

Paired reads obtained from Macrogen were analyzed using our in-house pipeline for metagenomics sequence processing—Metabakery (in preparation). Metabakery is a re-implementation of the BioBakery [52] workflow using (https://huttenhower.sph.harvard.edu/kneaddata/, accessed on 7 April 2022) for quality control, MetaPhlAn [53] for taxonomy analysis (bacteria, archaea, fungi, protozoa, and viruses), and HUMAn3 [53] for functional genes, enzymatic reactions, and metabolic pathways determination. Additionally, the MelonnPan was used for the prediction of metabolites. Metabakery is implanted as a singularity image and prepared on high computing performance clusters. The analyses running MetaBakery were performed on a dual Xeon system with 32 CPU cores (64 hyperthreads), 512 GB RAM and 6 TB SATA hard disc at the Faculty of Electrical Engineering, University of Ljubljana.

### 3.5. Characterization of Fecal Samples: Bristol Stool Scale, Metabolites, pH, MWI

Fecal samples were analyzed for a number of parameters as previously described and as follows [35]: Bristol stool scale (BSS) [123], water content, pH [124], total soluble organic carbon (TSOC), short-chain fatty acids (SCFA) [125], reducing sugars (Carbohydrate determination with 4-hydroxybenzoic acid hydrazide (PAHBAH)) [126], molecular weight, and dissolved organic carbon complexity using molecular weight indices [127,128]. In addition, fecal piercing strengths, as described before, were used as previously described [39].

### 3.6. Statictics and Machine Learning

#### 3.6.1. Statistics

First, the software PAST [129] was used for PERMANOVA. All obtained data matrices (NMR metabolomes—identified fecal and urinary metabolites at micromolar concentrations, microbial taxonomy, gene families, enzymatic reactions, metabolic pathways and predicted microbial metabolites) were analyzed in the same way. Each determined parameter was analyzed in three different ways as previously described [35,36,37,38,56]: (i) by dividing the measured concentration by the concentration of all metabolites in that sample; (ii) Box-Cox; or (iii) log(x + 1) transformed. The significance of the metabolic differences and microbial entities between the different sample groups were tested using ANOSIM, and NP-MANOVA, and expressed as the overlap in the non-metric multidimensional scaling (nm-MDS) trait space (using Euclidean distance measures). The stress function was used to select the dimensionality reduction, while Shepard’s plots were used to describe the correspondence between the target values and the obtained ranks. In addition, PCoA and PCA were performed on metagenomic data. Benjamini–Hochberg significance correction for multiple comparisons was used as previously described [130].

Second, for MetaboAnalyst [40], a log or cube root transformation was used in conjunction with mean or Pareto scaling as implemented in MetaboAnalyst, followed by supervised classification using the partial least squares discriminant analysis (PLSDA) method, random forest (RF), and pathway enrichment analysis. The PLSDA results were cross-validated with a caret package implemented in MeatboAnalyst. The major metabolites identified by PLSDA were determined according to the variable importance in projection (VIP). The randomForest package implemented in MetaboAnalyst was used for supervised classification between different groups of interest. The main features defined by RF were ordered according to the mean decrease in classification accuracy. Hierarchical clustering was performed according to the VIP scores to obtain a heat map representing the differences in metabolic profiles between samples and groups. Euclidean distance, Pearson’s correlation and Spearman’s correlation were used as similarity measures and Ward’s linkage was used as a clustering algorithm. MetaboAnalyst and gplot were used to generate graphs.

KEGG libraries for human metabolic pathways were used for metabolic pathway and enrichment analysis. For topological analysis, the globaltest analysis method and relative Betweenness centrality were used. Significant pathways were determined using the raw *p*-value, Holm–Bonferroni *p*-adjusted value, and adjusted *p*-value using the false discovery rate. The effect of pathways was calculated using the pathway topology analysis.

Metabolite Set Enrichment (MSEA) was used to identify biologically significant patterns between quantitative metabolome data from different groups. The names of compounds in Human Metabolome Database (HMDB) were used for linkage to the KEGG database. Enrichment analysis was performed using the globaltest package implemented in MetaboAnalyst. The enrichment ratio was calculated by dividing observed hits and expected hits.

#### 3.6.2. JADBIO Auto Machine Learning

Just Add Data Bio (JADBIO), a web-based machine learning platform for analyzing potential biomarkers [57], was used to search for biomarkers. The JADBIO platform was developed for predictive modeling and providing high-quality predictive models for diagnostics using state-of-the-art statistical and machine learning methods. Personal analytic biases and methodological statistical errors were eliminated from the analysis by autonomously exploring different settings in the modeling steps, resulting in more convincing discovered features to distinguish between different groups. JADBIO with extensive tuning effort and six CPUs was used to model different dataset choices in addition to the features observed in samples of all groups from different projects by splitting the total data into a training set and a test set in a 70:30 ratio. The training set was used to train the model and the test set was used to evaluate the model [39,56].

To assess the classification of the model, a receiver-operating characteristic curve (ROC curve) was constructed for all studied groups, plotting the true-positive rate (sensitivity) against the false-positive rate (1-specificity). Individual conditional expectation plots (ICE) showed the nature of the contribution of each feature characteristic to the model. All obtained models can be run locally using a Java executor.

## Data Availability

The data underlying this study are available in electronic Supplementary Materials.

## Supplementary Materials

The following supporting information can be downloaded at: https://www.mdpi.com/article/10.3390/metabo12060536/s1, ESM1: ESM 1; ESM 2: Metabolites identified in urinary samples; ESM 3: Metabolites identified in fecal samples; ESM 4: Models with instructions for local running.
