# Supplementary material for: Urine and Fecal 1H-NMR Metabolomes Differ Significantly between Pre-Term and Full-Term Born Physically Fit Healthy Adult Males
Source: Metabolites. 2022 Jun 10;12(6):536. doi: 10.3390/metabo12060536 (PMC9228004; doi:10.3390/metabo12060536)
Supplement: Supplementary file 1 [file metabolites-12-00536-s001.zip › ESM1.metabolites-1728434-supplementary.R1.x4.pdf]

Electronic Supplementary Material

Urine and fecal 1H-NMR metabolomes differ significantly between pre-term and full-term born physically fit healthy adult males

Leon Deutsch [1], Tadej Debevec [2,3], Gregoire P. Millet [2,4], Damjan Osredkar [5,6], Simona Opara[1], Robert Šket[7], Boštjan Murovec[8], Minca Mramor[9], Janez Plavec[10] and Blaz Stres [1,3,11*]

[1] University of Ljubljana, Biotechnical Faculty, Department of Animal Science, Ljubljana, Slovenia; leon.deutsch@bf.uni-lj.si
[2] Faculty of Sports, University of Ljubljana, Ljubljana, Slovenia. tadej.debevec@fsp.uni-lj.si
[3] Jožef Stefan Institute, Department of Automation, Biocybernetics and Robotics, Ljubljana, Slovenia
[4] Institute of Sport Sciences, University of Lausanne, Lausanne, Switzerland. gregoire.millet@unil.ch
[5] Department of Pediatric Neurology, University Children's Hospital, University Medical Centre Ljubljana, Slovenia; damjan.osredkar@kclj.si
[6] Faculty of Medicine, University of Ljubljana, Slovenia
[7] Institute for Special Laboratory Diagnostics, University Medical Centre Ljubljana, University Children's Hospital, Ljubljana, Slovenia
[8] University of Ljubljana, Faculty of electrical engineering, Jamova 2, SI-1000 Ljubljana; bostjan.murovec@fe.uni-lj.si
[9] Department of Infectious diseases, University Medical Centre Ljubljana, Slovenia; minca.mramore@kclj.si
[10] National Institute of Chemistry, NMR Center, Ljubljana, Slovenia; janez.plavec@ki.si
[11] University of Ljubljana, Faculty of Civil and Geodetic Engineering, Institute of Sanitary Engineering, Ljubljana, Slovenia
* Correspondence: blaz.stres@bf.uni-lj.si; Tel.: +38641567633

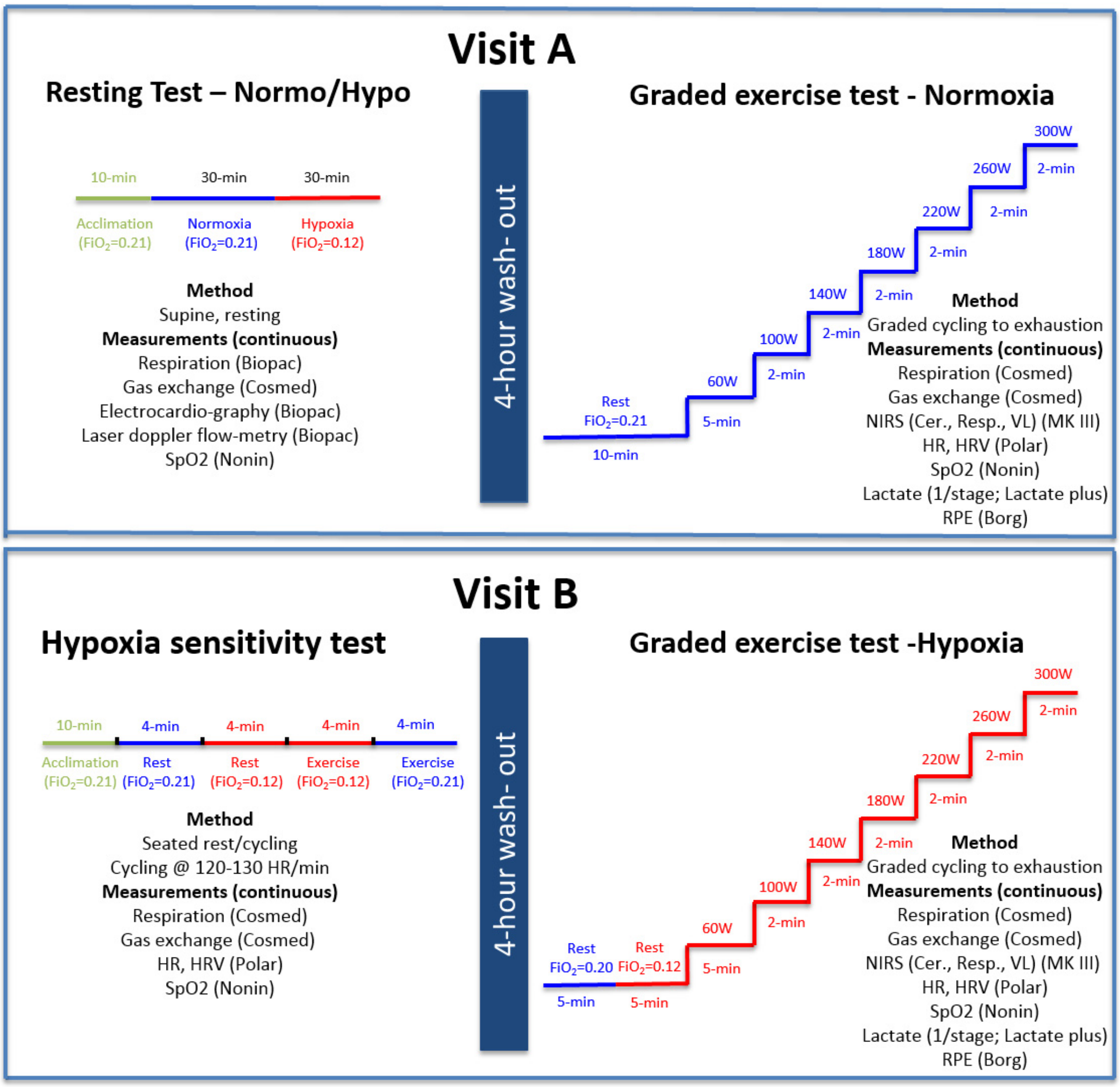

Figure S1: The outline of the PreTerm project. The participants visited the lab on two occasions in a randomised fashion (i.e. visit A & visit B). Collectively, they performed four tests:
1) **Resting normo/hypo tes**t - this was suggested by our colleagues from the Medical faculty to assess different physiological parameters during resting state in normoxia and hypoxia.
2) **Hypoxia sensitivity test** (i.e. Richalet test) to assess hypoxia sensitivity and derive HVR.
3) **Graded exercise test in normoxia**
4) **Graded exercise test in hypoxia**
Both graded exercise tests have a relatively gradual power increases (40W/4 min). The participants were blinded (as to normo/hypo) during all tests [1,2].

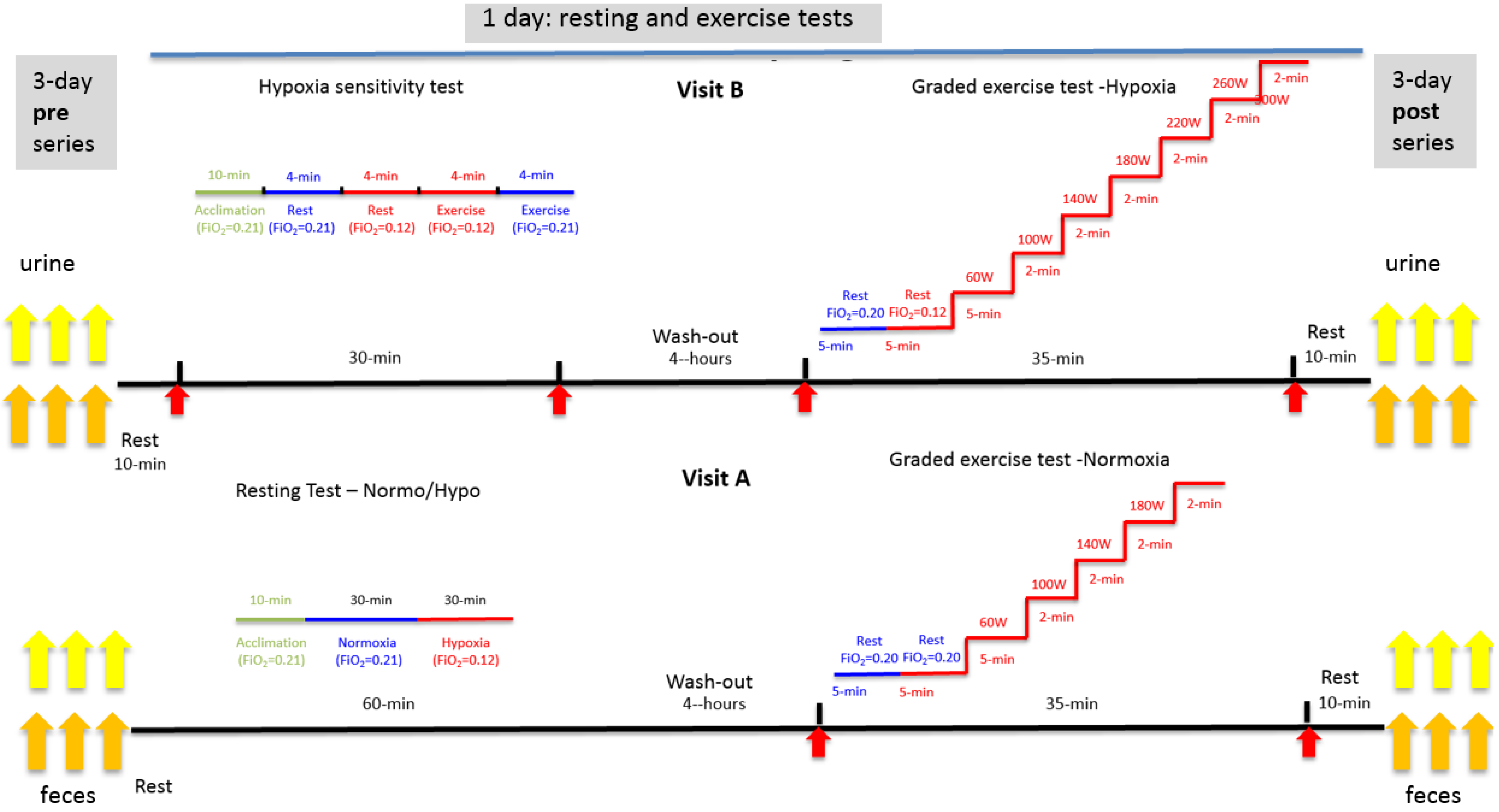

Figure S2: Sampling procedures of the PreTerm project. The inset designates the detailed procedures utilized for analyses of air and blood samples reported before [1,2]. In this study focused on the 3-day series before and after resting and exercise tests in normoxia and hypoxia.

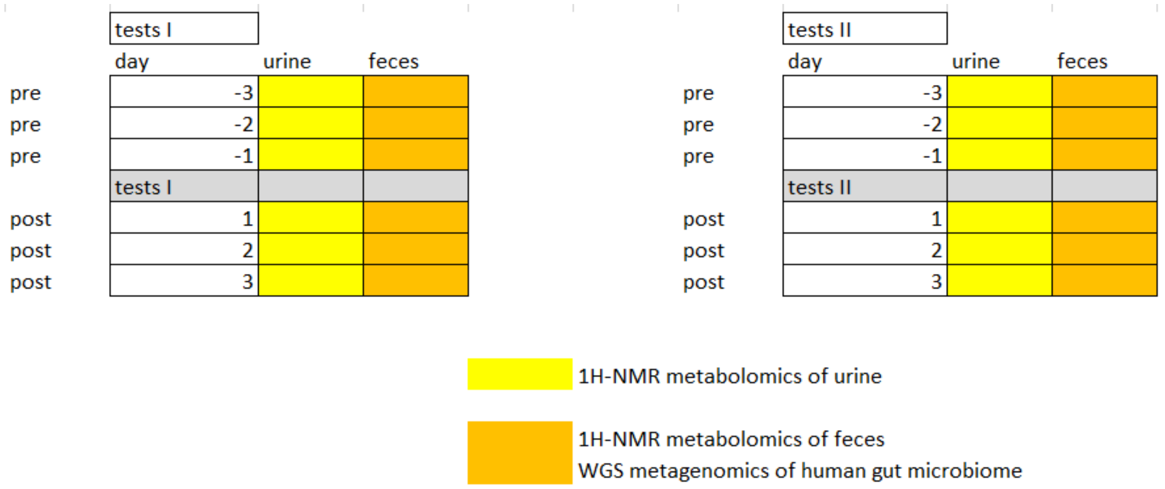

Figure S3: Schematic representation of 3-day sampling series utilized in this study to detected significant differences in the measured metabolomics and metagenomics datasets collected from urine and fecal samples. Every participant collected 12 fecal and 12 urine samples (three before and three after normoxic tests, next to additional three before and three after hypoxic tests).

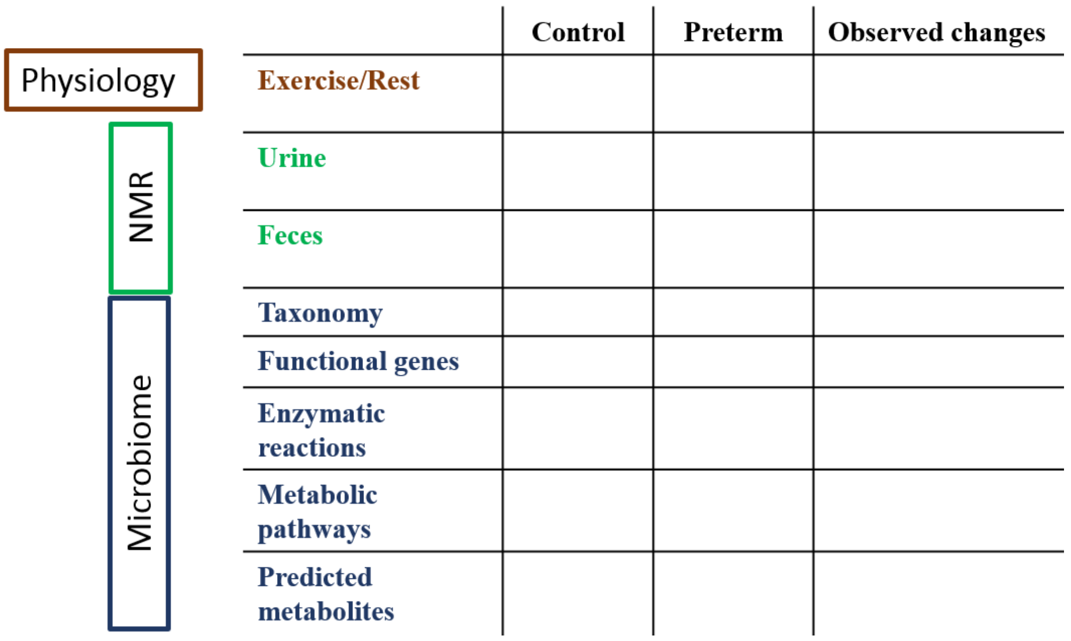

Figure S4: A schematic representation of the explored data matrices between the two groups of participants.

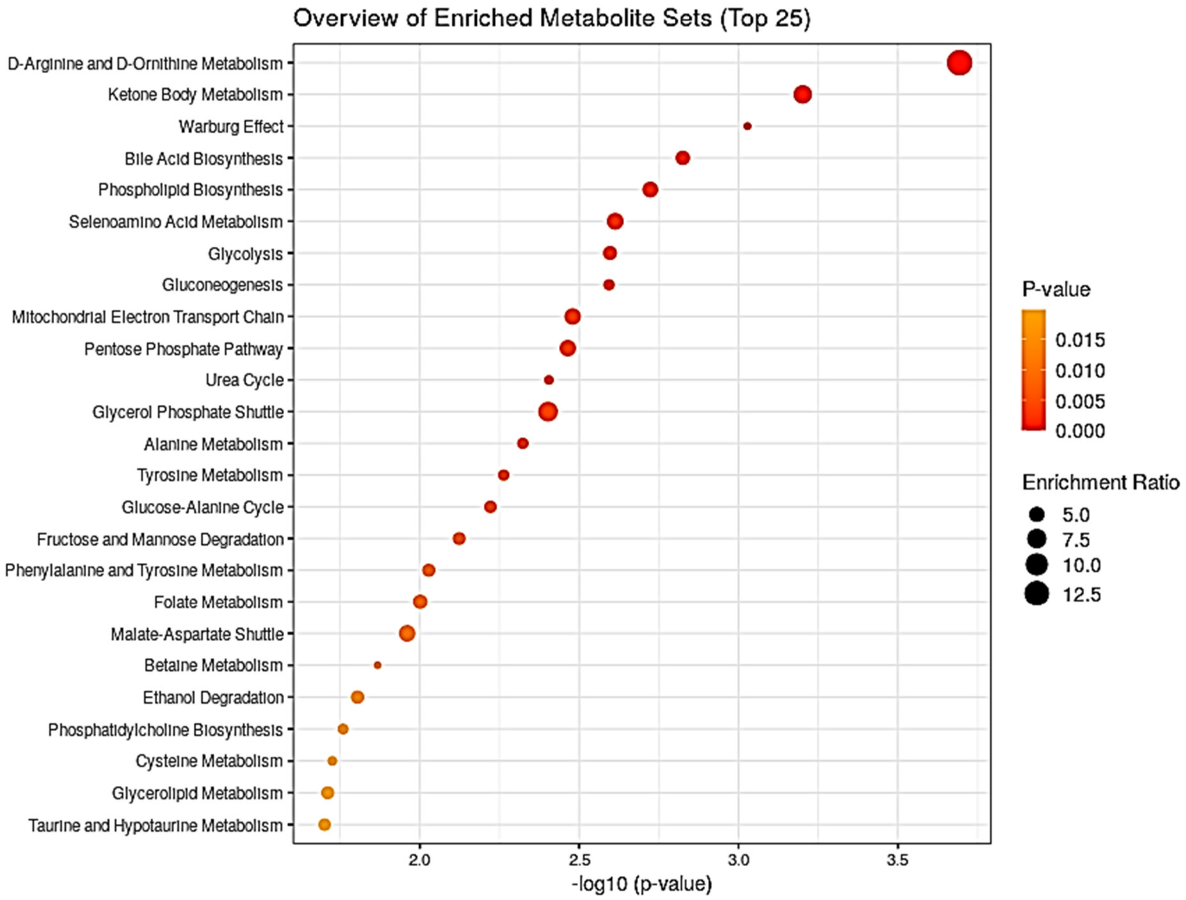

Figure S5: The most enriched metabolic pathways associated with metabolism based on urinary metabolomes.

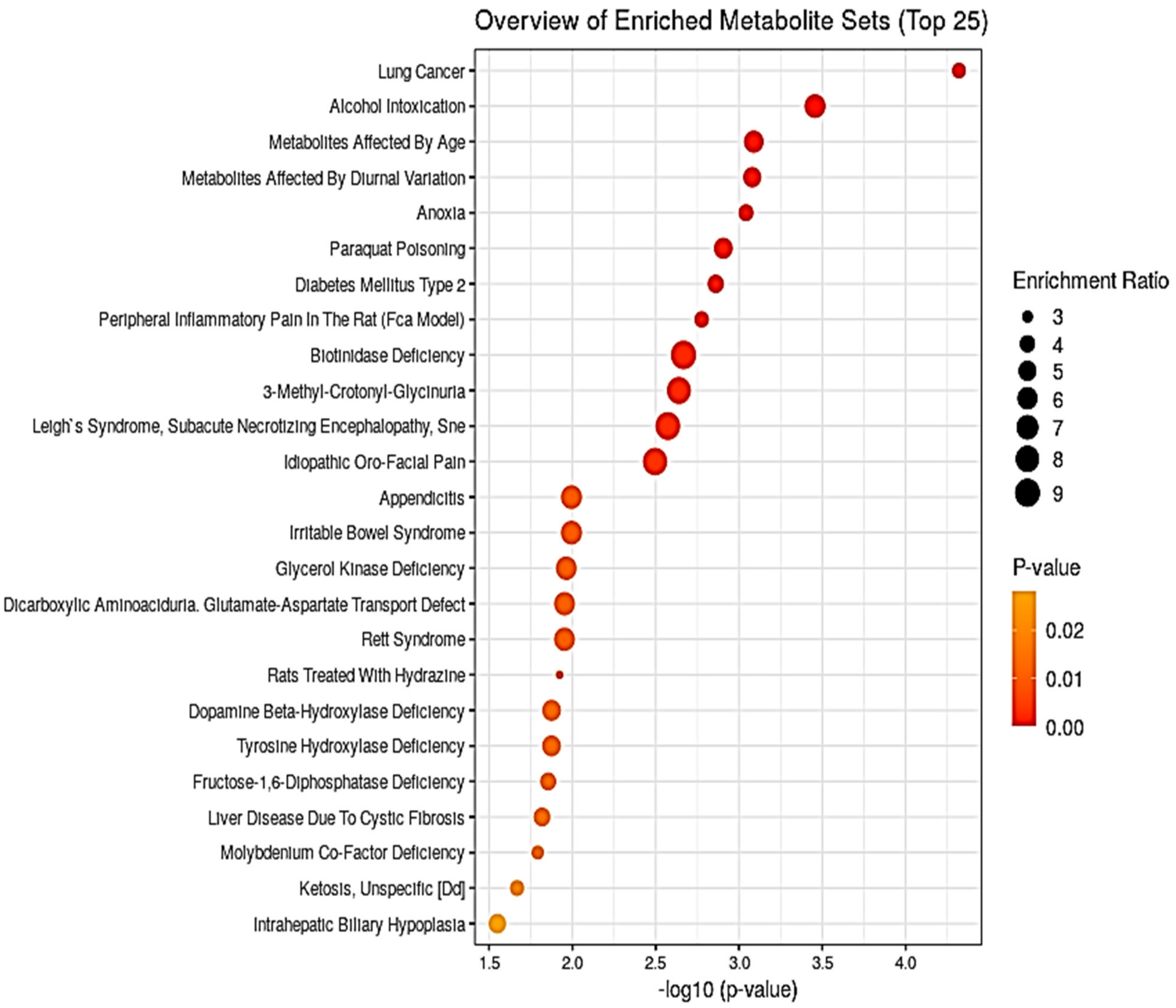

Figure S6: The most enriched metabolic pathways associated with disease based on urinary metabolomes.

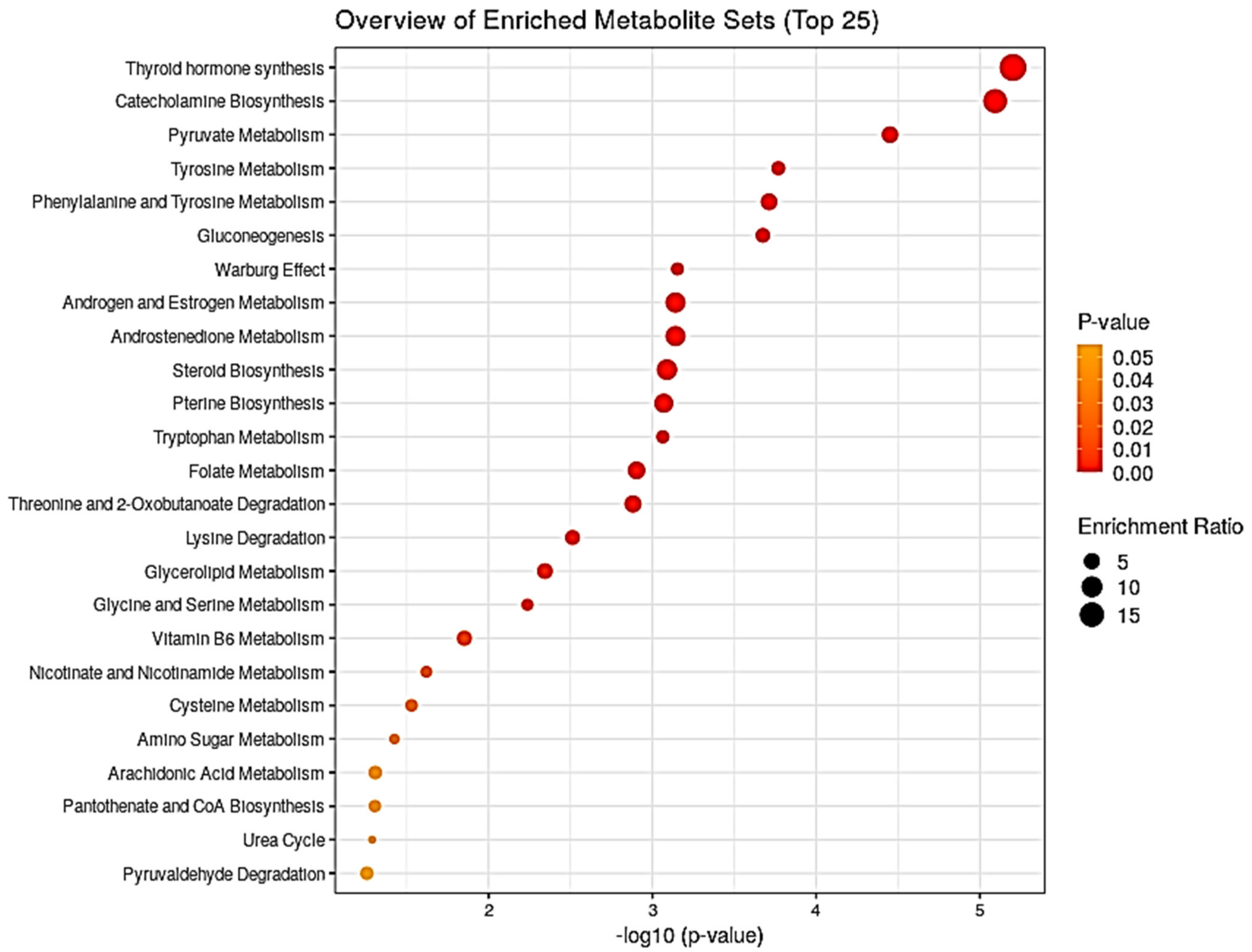

Figure S7: The most enriched metabolic pathways associated with metabolism based on fecal metabolomes.

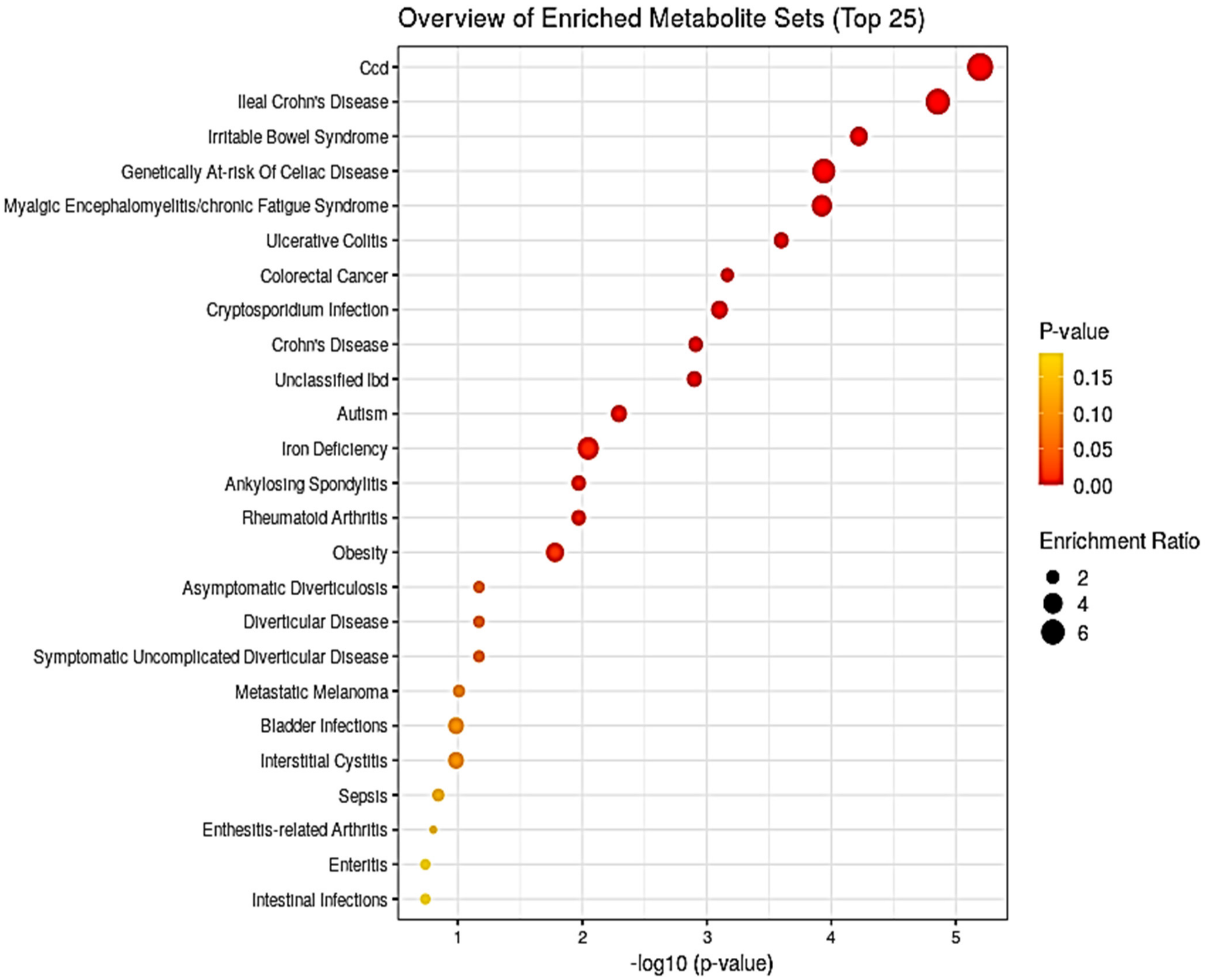

Figure S8: The most enriched metabolic pathways associated with disease based on fecal metabolomes.

a) Kingdom abundance b) Shannon diversity

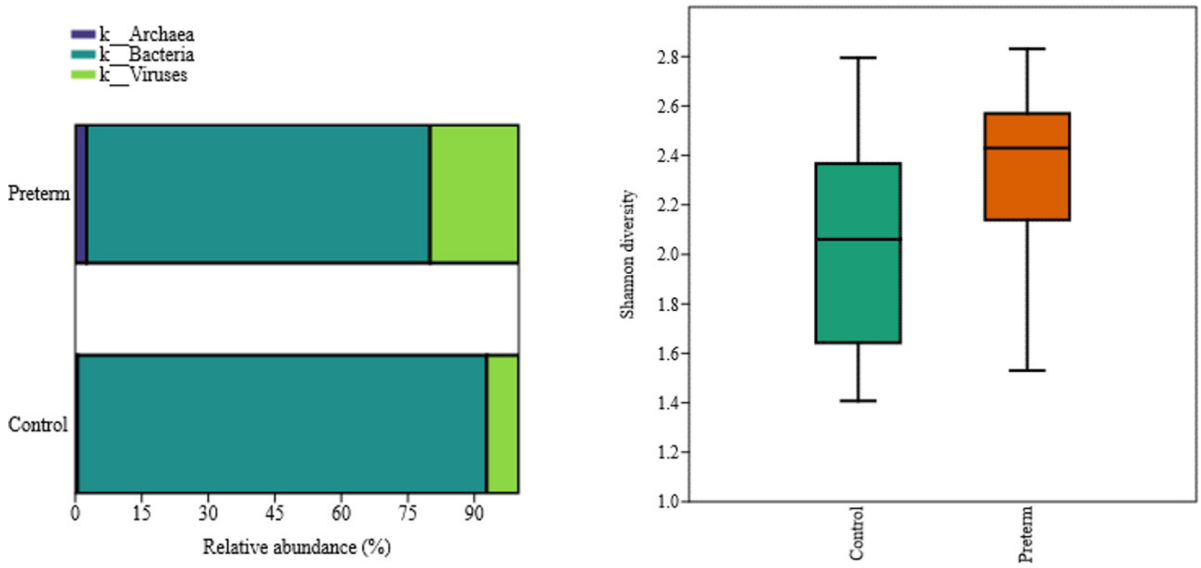

Figure S9. Abundance of three different kingdoms in preterm and full term group based on fecal metagenomics (a). Shannon diversity was significantly increased in Preterm group (b).

b) RXN-15378

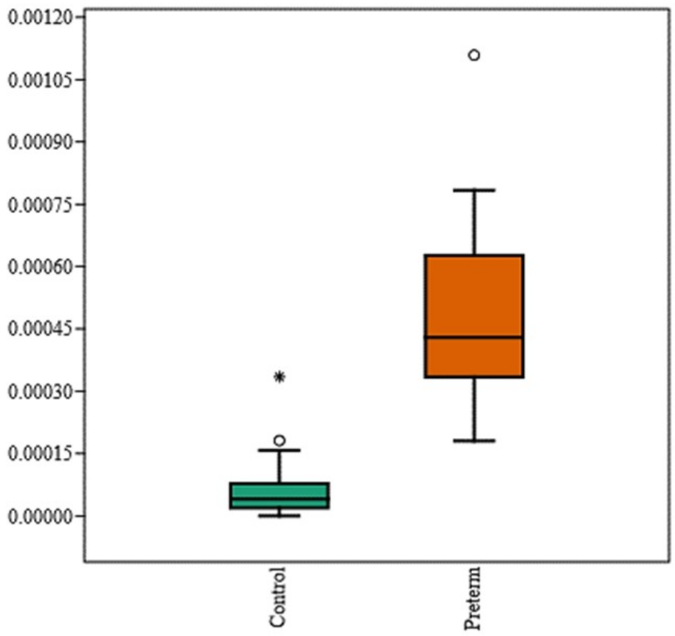

Figure S10. Box plots presenting the relative abundance of RXN-15378 in preterm (orange) and control (group). This enzymatic reaction is involved in the production of succinate.

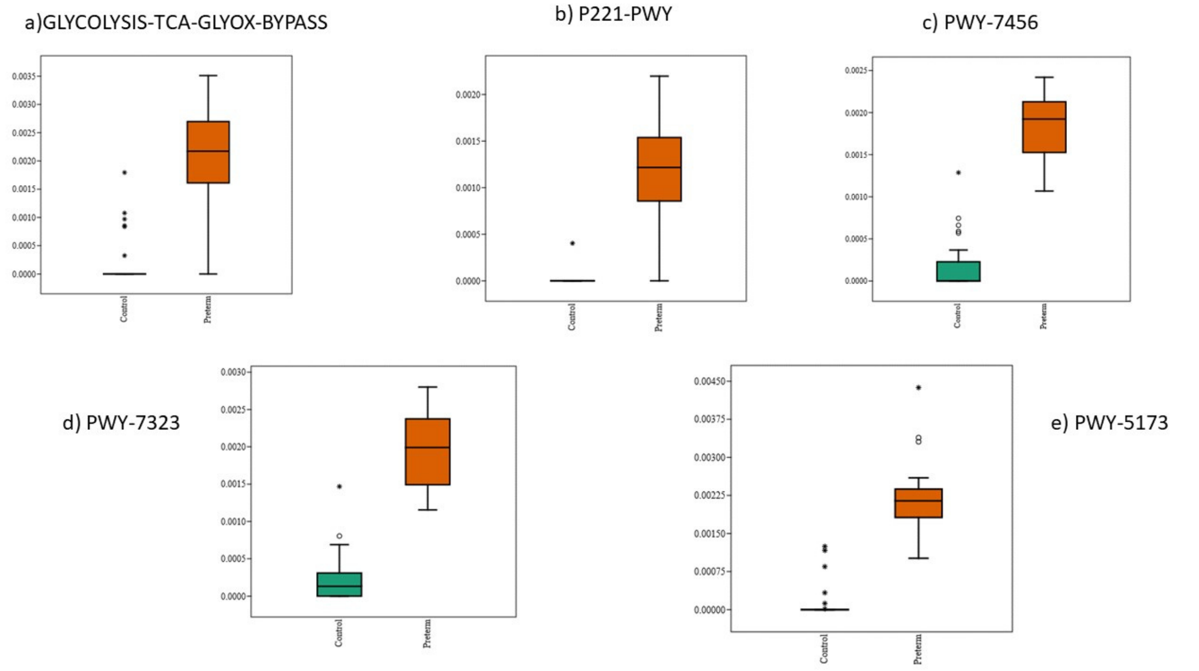

Figure S11. Selected metabolic pathways for differentiation of preterm (orange= and control (green) group.

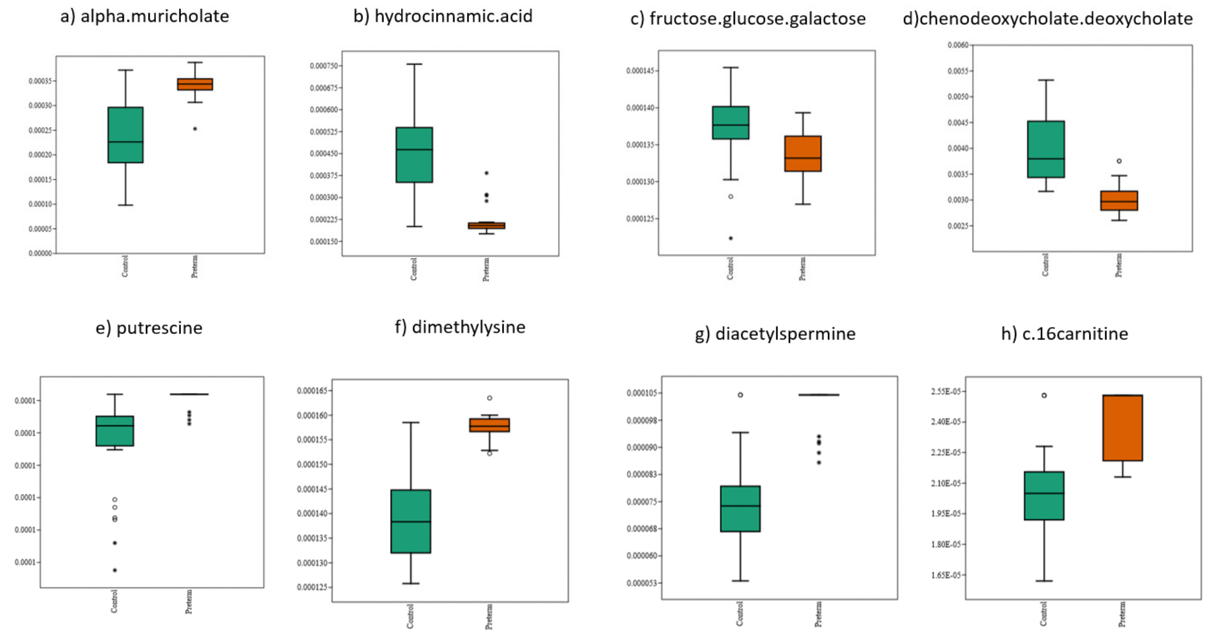

Figure S12. Selected predicted metabolites associated with human gut microbiota in control (green) and preterm (orange) groups.

		Control	Preterm	Observed changes
Physiology	Exercise/Rest	●	▲	Preterm significantly different from full-term and different responses to rest and some to exercise
NMR	Urine	●	▲	Preterm significantly different from full-term at the level of urine metabolomes
NMR	Feces	●	▲	Preterm significantly different from full-term at the level of fecal metabolomes
Microbiome	Taxonomy	●	●	No characteristic change in microbiome taxonomy
Microbiome	Functional genes	●	●	No characteristic change in microbiome functional genes
Microbiome	Enzymatic reactions	●	▲	Distinct use of enzymatic reactions
Microbiome	Metabolic pathways	●	▲	Distinct use of metabolic pathways
Microbiome	Predicted metabolites	●	▲	Distinct use of predicted metabolites

Figure S13: A summary of observed changes at various information levels showing that significant differences exist between the preterm and full-term adult urine metabolomes, fecal metabolomes and microbial metabolic reactions and pathways. Taken together, these results show that host and its microbiome behave measurably different in healthy physically fit young males in comparison to matched full-term controls.

Table S1. Baseline characteristics of PreTerm project participants [2].

Variable	Full-term (n = 15)		Pre-term (n = 22)	
	Mean	SD	Mean	SD
Age (years)	22	2	21	2
Body mass (kg)	73	6	69	7
Height (cm)	180	5	175***	7
BMI ($kg.m^{-2}$)	23	2	23	3
Gestational age (weeks)	39	2	29***	3
$\dot{V}O_2max$ ($mL.kg^{-1}.min^{-1}$)	52	5	48	6

Table S2. The number of detected urine and fecal metabolites comparing full-term and preterm participants of the PreTerm study. Numbers in bold represent the observed albeit not significant differences in the number of metabolites within the preterm group between the Before-Hyp and After-Norm, and call for further exploration of potentially larger adaptability of preterm participants to oxidative challenges.

		TERM				Preterm			
		Hyp		Nor		Hyp		Nor	
		Before	After	Before	After	Before	After	Before	After
urine	Average	132.1	139.8	138.9	132.0	***121.7***	116.7	127.2	***139.5***
	SD	30.6	15.7	15.7	27.4	***32.7***	36.5	39.1	***25.6***
feces	Average	82.3	78.2	80.8	81.2	***81.9***	84.1	82.5	***89.5***
	SD	20.7	29.9	23.8	21.2	***21.0***	21.2	23.0	***11.9***

Table S3. Detected metabolic pathways in urine.

Metabolic pathway	Total Cmpd	Hits	Statistic Q	Expected Q	Raw p	Holm p	FDR
D-Arginine and D-Ornithine Metabolism	11	1	6.95670	0.51813	0.00020	0.01943	0.01943
Ketone Body Metabolism	13	4	3.61810	0.51813	0.00063	0.05972	0.03000
Warburg Effect	58	17	1.69810	0.51813	0.00094	0.08812	0.03000
Bile Acid Biosynthesis	65	7	2.44720	0.51813	0.00150	0.13909	0.03059
Phospholipid Biosynthesis	29	4	2.79900	0.51813	0.00189	0.17399	0.03059
Selenoamino Acid Metabolism	28	5	3.08090	0.51813	0.00244	0.22175	0.03059
Glycolysis	25	8	2.35670	0.51813	0.00253	0.22768	0.03059
Gluconeogenesis	35	12	1.91410	0.51813	0.00255	0.22768	0.03059
Mitochondrial Electron Transport Chain	19	5	2.97400	0.51813	0.00331	0.29169	0.03168
Pentose Phosphate Pathway	29	5	2.98080	0.51813	0.00343	0.29864	0.03168
Urea Cycle	29	12	1.76740	0.51813	0.00393	0.33830	0.03168
Glycerol Phosphate Shuttle	11	2	4.09630	0.51813	0.00396	0.33830	0.03168
Alanine Metabolism	17	8	1.93380	0.51813	0.00475	0.39897	0.03507
Tyrosine Metabolism	72	16	1.91970	0.51813	0.00545	0.45266	0.03740
Glucose-Alanine Cycle	13	6	2.07490	0.51813	0.00600	0.49220	0.03842
Fructose and Mannose Degradation	32	9	2.13370	0.51813	0.00752	0.60933	0.04514
Phenylalanine and Tyrosine Metabolism	28	7	2.18100	0.51813	0.00937	0.74969	0.05292
Folate Metabolism	29	5	2.47720	0.51813	0.00997	0.78750	0.05317
Malate-Aspartate Shuttle	10	4	3.00320	0.51813	0.01096	0.85471	0.05537
Betaine Metabolism	21	9	1.69700	0.51813	0.01356	1.00000	0.06511
Ethanol Degradation	19	4	2.2121	0.51813	0.015674	1	0.071652
Phosphatidylcholine Biosynthesis	14	6	1.85930	0.51813	0.01740	1.00000	0.07593
Cysteine Metabolism	26	7	1.76290	0.51813	0.01881	1.00000	0.07638
Glycerolipid Metabolism	25	5	2.16140	0.51813	0.01944	1.00000	0.07638
Taurine and Hypotaurine Metabolism	12	2	2.08390	0.51813	0.01989	1.00000	0.07638
Nucleotide Sugars Metabolism	20	9	1.32390	0.51813	0.02630	1.00000	0.09573
Phosphatidylethanolamine Biosynthesis	12	4	1.92190	0.51813	0.02783	1.00000	0.09573
Glutamate Metabolism	49	16	1.17350	0.51813	0.02792	1.00000	0.09573
Arginine and Proline Metabolism	53	16	1.13860	0.51813	0.03285	1.00000	0.10875
Glycine and Serine Metabolism	59	25	0.96882	0.51813	0.03446	1.00000	0.11028
Aspartate Metabolism	35	13	1.15460	0.51813	0.03804	1.00000	0.11779
Methionine Metabolism	43	15	1.07280	0.51813	0.04148	1.00000	0.12007
Citric Acid Cycle	32	12	1.15480	0.51813	0.04472	1.00000	0.12007
Butyrate Metabolism	19	5	1.56920	0.51813	0.04486	1.00000	0.12007
Amino Sugar Metabolism	33	9	1.10770	0.51813	0.04614	1.00000	0.12007
Tryptophan Metabolism	60	19	1.25150	0.51813	0.04728	1.00000	0.12007
Ammonia Recycling	32	13	1.07160	0.51813	0.04730	1.00000	0.12007
Lysine Degradation	30	6	1.29900	0.51813	0.04753	1.00000	0.12007

Table S4. Detected metabolic pathways and urine related diseases.

Disease	Total Cmpd	Hits	Statistic Q	Expected Q	Raw p	Holm p	FDR
Lung Cancer	32	29	1.85450	0.51813	0.00005	0.01100	0.01100
Alcohol Intoxication	4	4	3.38020	0.51813	0.00035	0.08007	0.04021
Metabolites Affected By Age	12	12	3.00170	0.51813	0.00082	0.18596	0.04175
Metabolites Affected By Diurnal Variation	6	6	2.62390	0.51813	0.00083	0.18898	0.04175
Anoxia	9	8	2.02080	0.51813	0.00091	0.20511	0.04175
Paraquat Poisoning	7	7	2.77860	0.51813	0.00124	0.27933	0.04530
Diabetes Mellitus Type 2	12	8	2.26770	0.51813	0.00138	0.30880	0.04530
Peripheral Inflammatory Pain In The Rat (Fca Model)	10	9	1.95070	0.51813	0.00168	0.37355	0.04816
Biotinidase Deficiency	3	1	4.79750	0.51813	0.00215	0.47780	0.05275
3-Methyl-Crotonyl-Glycinuria	4	2	4.31570	0.51813	0.00229	0.50687	0.05275
Leigh`s Syndrome Subacute Necrotizing Encephalopathy	1	1	4.59970	0.51813	0.00267	0.58831	0.05591
Idiopathic Oro-Facial Pain	2	1	4.43930	0.51813	0.00319	0.69846	0.06113
Appendicitis	1	1	3.39420	0.51813	0.01013	1.00000	0.15084
Irritable Bowel Syndrome	1	1	3.39420	0.51813	0.01013	1.00000	0.15084
Glycerol Kinase Deficiency	1	1	3.32930	0.51813	0.01088	1.00000	0.15084
Dicarboxylic Aminoaciduria. Glutamate-Aspartate Transport Defect	3	1	3.30770	0.51813	0.01115	1.00000	0.15084
Rett Syndrome	2	1	3.30770	0.51813	0.01115	1.00000	0.15084
Rats Treated With Hydrazine	9	9	1.45000	0.51813	0.01193	1.00000	0.15248
Dopamine Beta-Hydroxylase Deficiency	5	2	2.77010	0.51813	0.01334	1.00000	0.15298
Tyrosine Hydroxylase Deficiency	3	2	2.77010	0.51813	0.01334	1.00000	0.15298

Table S5. Detected metabolic pathways in feces.

Metabolic pathway	Total Cmpd	Hits	Statistic Q	Expected Q	Raw p	Holm p	FDR
Thyroid hormone synthesis	13	1	10.256	0.52632	6.29E-06	0.000604	0.000388
Catecholamine Biosynthesis	20	3	7.5306	0.52632	8.07E-06	0.000767	0.000388
Pyruvate Metabolism	48	11	2.9013	0.52632	3.54E-05	0.003327	0.001133
Tyrosine Metabolism	72	16	1.9207	0.52632	0.00017	0.015808	0.003381
Phenylalanine and Tyrosine Metabolism	28	9	3.0289	0.52632	0.000194	0.017829	0.003381
Gluconeogenesis	35	11	2.1448	0.52632	0.000211	0.019228	0.003381
Warburg Effect	58	17	1.6491	0.52632	0.000703	0.063235	0.006898
Androgen and Estrogen Metabolism	33	3	4.8509	0.52632	0.000722	0.064251	0.006898
Androstenedione Metabolism	24	3	4.8509	0.52632	0.000722	0.064251	0.006898
Steroid Biosynthesis	48	2	5.1195	0.52632	0.000814	0.070824	0.006898
Pterine Biosynthesis	29	3	4.2018	0.52632	0.000851	0.073204	0.006898
Tryptophan Metabolism	60	19	1.7065	0.52632	0.000862	0.073294	0.006898
Folate Metabolism	29	4	3.4721	0.52632	0.001249	0.10494	0.009
Threonine and 2-Oxobutanoate Degradation	20	4	3.2252	0.52632	0.001313	0.10893	0.009
Lysine Degradation	30	6	2.254	0.52632	0.00307	0.2517	0.019645
Glycerolipid Metabolism	25	4	2.5627	0.52632	0.004524	0.36647	0.027146
Glycine and Serine Metabolism	59	24	1.4655	0.52632	0.005786	0.46287	0.032673
Vitamin B6 Metabolism	20	2	2.3409	0.52632	0.014039	1	0.074874
Nicotinate and Nicotinamide Metabolism	37	9	1.403	0.52632	0.023947	1	0.121
Cysteine Metabolism	26	7	1.5637	0.52632	0.029509	1	0.14164
Amino Sugar Metabolism	33	8	1.1987	0.52632	0.037498	1	0.17142
Arachidonic Acid Metabolism	69	2	1.8317	0.52632	0.049112	1	0.20527
Pantothenate and CoA Biosynthesis	21	4	1.5395	0.52632	0.049389	1	0.20527

Table S6. Detected metabolic pathways and fecal related diseases.

	Total Cmpd	Hits	Statistic Q	Expected Q	Raw p	Holm p	FDR
Ccd	17	5	4.0642	0.52632	6.38E-06	0.000242	0.000242
Ileal Crohn's Disease	19	6	3.437	0.52632	1.40E-05	0.000516	0.000265
Irritable Bowel Syndrome	186	110	1.796	0.52632	6.01E-05	0.002164	0.000761
Genetically At-risk Of Celiac Disease	5	5	3.0775	0.52632	0.000115	0.004017	0.000906
Myalgic Encephalomyelitis/chronic Fatigue Syndrome	22	21	2.3309	0.52632	0.000119	0.004053	0.000906
Ulcerative Colitis	609	202	1.279	0.52632	0.000252	0.00833	0.001599
Colorectal Cancer	1653	374	1.0738	0.52632	0.000684	0.0219	0.003715
Cryptosporidium Infection	29	19	1.6033	0.52632	0.000792	0.024552	0.003762
Crohn's Disease	522	216	1.2162	0.52632	0.001229	0.036855	0.004794
Unclassified Ibd	84	58	1.292	0.52632	0.001262	0.036855	0.004794
Autism	204	54	1.4493	0.52632	0.005077	0.14215	0.017537
Iron Deficiency	50	4	2.5774	0.52632	0.008974	0.2423	0.028418
Ankylosing Spondylitis	21	20	1.2335	0.52632	0.010693	0.27801	0.029023
Rheumatoid Arthritis	42	40	1.2335	0.52632	0.010693	0.27801	0.029023
Obesity	14	6	1.8613	0.52632	0.016567	0.39761	0.04197

Table S7. The statistical significance in calculated diversity indices.

Diversity index	p	t
ace	**1.20E-15**	**11.092**
bergerparker	**1.10E-20**	**14.695**
boneh	**5.10E-21**	**14.948**
bstick	**2.60E-19**	**13.695**
chao	**2.10E-24**	**17.721**
coverage	**1.40E-81**	**209.94**
efron	**1.60E-04**	**4.0592**
geometric	**2.42E-18**	**12.952**
goodscoverage	**1.40E-81**	**209.94**
heip	**2.88E-35**	**29.331**
invsimpson	**3.93E-24**	**17.491**
jackknife	1.32E-01	1.528
logseries	**4.99E-25**	**18.276**
shannon	**1.01E-43**	**42.45**
shannoneven	**4.05E-52**	**60.785**
simpson	**9.76E-20**	**13.971**
simpsoneven	**4.18E-30**	**23.227**
smithwilson	**7.49E-24**	**17.252**
sobs	**2.50E-37**	**32.133**

Table S8. Matching metabolites identified in fecal samples by 1H-NMR and predicted by MelonPan.

Matching metabolites
azelate
cholate
citrulline
creatine
glutamate
hypoxanthine
inosine
malonate
nicotinate
pantothenate
phenylacetate
propionate
sebacate
taurine
thymine
uracil
xanthine

References

1. Debevec, T.; Pialoux, V.; Millet, G.P.; Martin, A.; Mramor, M.; Osredkar, D. Exercise Overrides Blunted Hypoxic Ventilatory Response in Prematurely Born Men. *Frontiers in physiology* **2019**, *10*, doi:10.3389/fphys.2019.00437.
2. Martin, A.; Millet, G.; Osredkar, D.; Mramor, M.; Faes, C.; Gouraud, E.; Debevec, T.; Pialoux, V. Effect of pre-term birth on oxidative stress responses to normoxic and hypoxic exercise. *Redox biology* **2020**, *32*, doi:10.1016/j.redox.2020.101497.
