# Supplementary material for: Urine and Fecal 1H-NMR Metabolomes Differ Significantly between Pre-Term and Full-Term Born Physically Fit Healthy Adult Males
Source: Metabolites. 2022 Jun 10;12(6):536. doi: 10.3390/metabo12060536 (PMC9228004; doi:10.3390/metabo12060536)
Supplement: Supplementary file 1 [file metabolites-12-00536-s001.zip › ESM4.models/Instructions for running a model on a local machine.pdf]

**Instructions for running a model on a local machine**

JADBIO allows the user to download a model and run it on a local machine.

To run our model locally, the user must meet the following requirements:

1. Java SE Development Kit version 17 (https://www.oracle.com/java/technologies/javase/jdk17-archive-downloads.html)

2. the Java executor (contained in model.zip - filename: jadbio-1.4.55-model-exe.jar)

3. created models based on enzymatic reactions, metabolic pathways and predicted microbial metabolites (contained in model.zip - filenames: ecs.bin, pathways.bin, PredictedMetabolites.bin)

After installing Java SE JDK, model.zip must be saved somewhere on the local machine. After saving model.zip, provided by the authors, the folder must be extracted (e.g. with WinZip, 7zip). The model must be executed with the command prompt (cmd) (Fig. 1, 2).

**Step 1**

Using the `cd path` command (Fig. 1), the user navigates to the same directory (e.g. Folder) that contains the model executor (.jar) and the model (.bin).

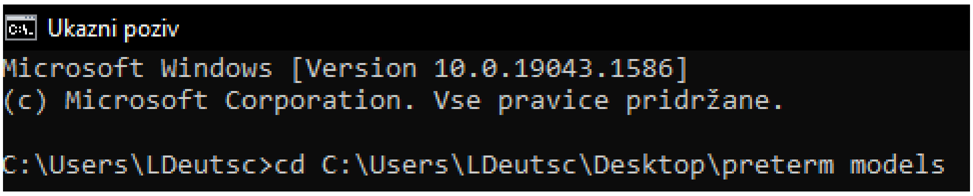


**Figure 1.** First command to navigate to the folder containing the model. In this case, we used the `cd C:\Users\LDeutsc\Desktop\preterm models` command because Executor and Models were in the preterm models folder on the desktop.

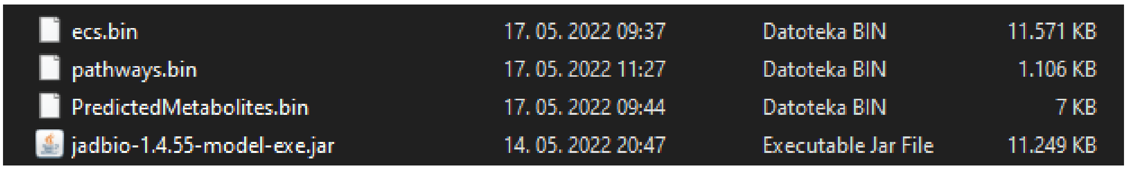


**Figure 2.** The two files needed for the overview of the model. Both files are contained in the model.zip folder.

**Step 2**

The next step is to preview the model using the following command:

```
Java --enable-preview -jar jadbio-1.4.55-model-exe.jar -n <NAME OF THE MODEL>.bin
```

This allows the user to get an overview of the model, key features and information about the analysis (which algorithm was used, version of JADBIO and other information about the model) (Fig. 3).

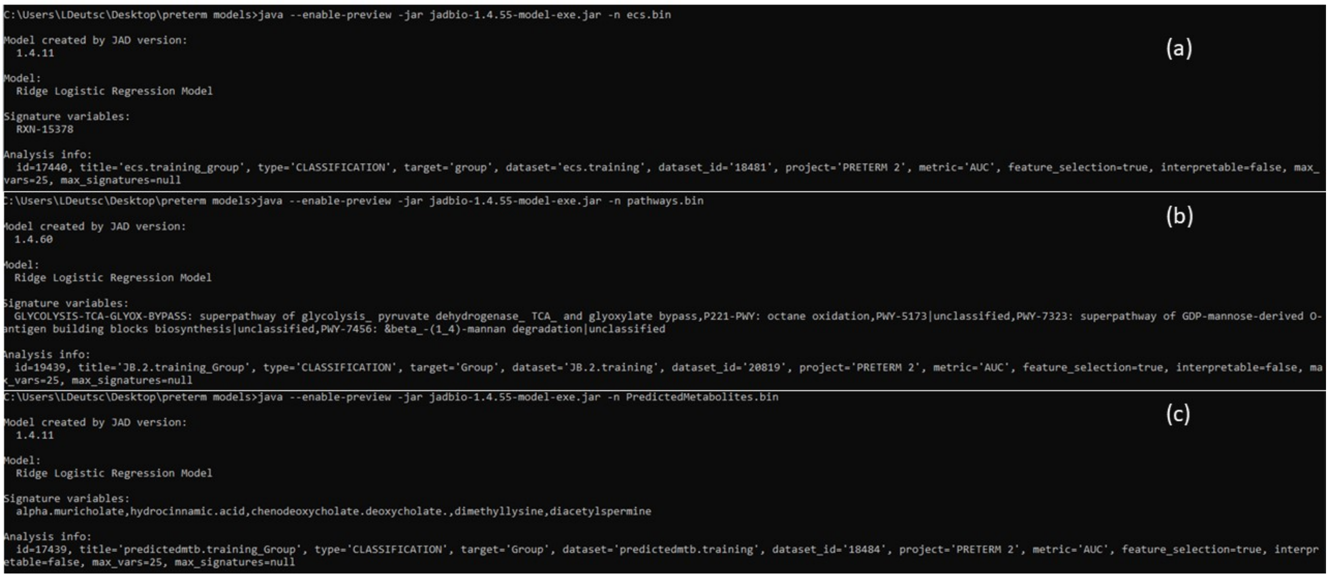

```
C:\Users\LDeutsc\Desktop\preterm models>java --enable-preview -jar jadbio-1.4.55-model-exe.jar -n ecs.bin

Model created by JAD version:
  1.4.11

Model:
  Ridge Logistic Regression Model

Signature variables:
  RXN-15378

Analysis info:
  id=17440, title='ecs.training_group', type='CLASSIFICATION', target='group', dataset='ecs.training', dataset_id='18481', project='PRETERM 2', metric='AUC', feature_selection=true, interpretable=false, max_
vars=25, max_signatures=null
```

(a)

```
C:\Users\LDeutsc\Desktop\preterm models>java --enable-preview -jar jadbio-1.4.55-model-exe.jar -n pathways.bin

Model created by JAD version:
  1.4.60

Model:
  Ridge Logistic Regression Model

Signature variables:
  GLYCOLYSIS-TCA-GLYOX-BYPASS: superpathway of glycolysis_ pyruvate dehydrogenase_ TCA_ and glyoxylate bypass,P221-PWY: octane oxidation,PWY-5173|unclassified,PWY-7323: superpathway of GDP-mannose-derived O-
antigen building blocks biosynthesis|unclassified,PWY-7456: &beta_-(1_4)-mannan degradation|unclassified

Analysis info:
  id=19439, title='JB.2.training_Group', type='CLASSIFICATION', target='Group', dataset='JB.2.training', dataset_id='20819', project='PRETERM 2', metric='AUC', feature_selection=true, interpretable=false, ma
x_vars=25, max_signatures=null
```

(b)

```
C:\Users\LDeutsc\Desktop\preterm models>java --enable-preview -jar jadbio-1.4.55-model-exe.jar -n PredictedMetabolites.bin

Model created by JAD version:
  1.4.11

Model:
  Ridge Logistic Regression Model

Signature variables:
  alpha.muricholate,hydrocinnamic.acid,chenodeoxycholate.deoxycholate.,dimethyllysine,diacetylspermine

Analysis info:
  id=17439, title='predictedmtb.training_Group', type='CLASSIFICATION', target='Group', dataset='predictedmtb.training', dataset_id='18484', project='PRETERM 2', metric='AUC', feature_selection=true, interpr
etable=false, max_vars=25, max_signatures=null
```

(c)

**Figure 3.** Overview of the models for (a) enzymatic reactions, (b) metabolic pathways and (c) predicted metabolites.

**Step 3**

To test the model, the user must prepare data. For the model to work properly, the output of HUMAnN 3.0 and MelonnPan must be used. The user needs to prepare the dataset as shown in Figure 4 and save it in a .csv document (comma separated values). Names of features must be prepared in a way, that is presented in preview of the model (section named Signature variables). After preparation, the prepared dataset must be saved in the same directory as the model executor and the model itself.

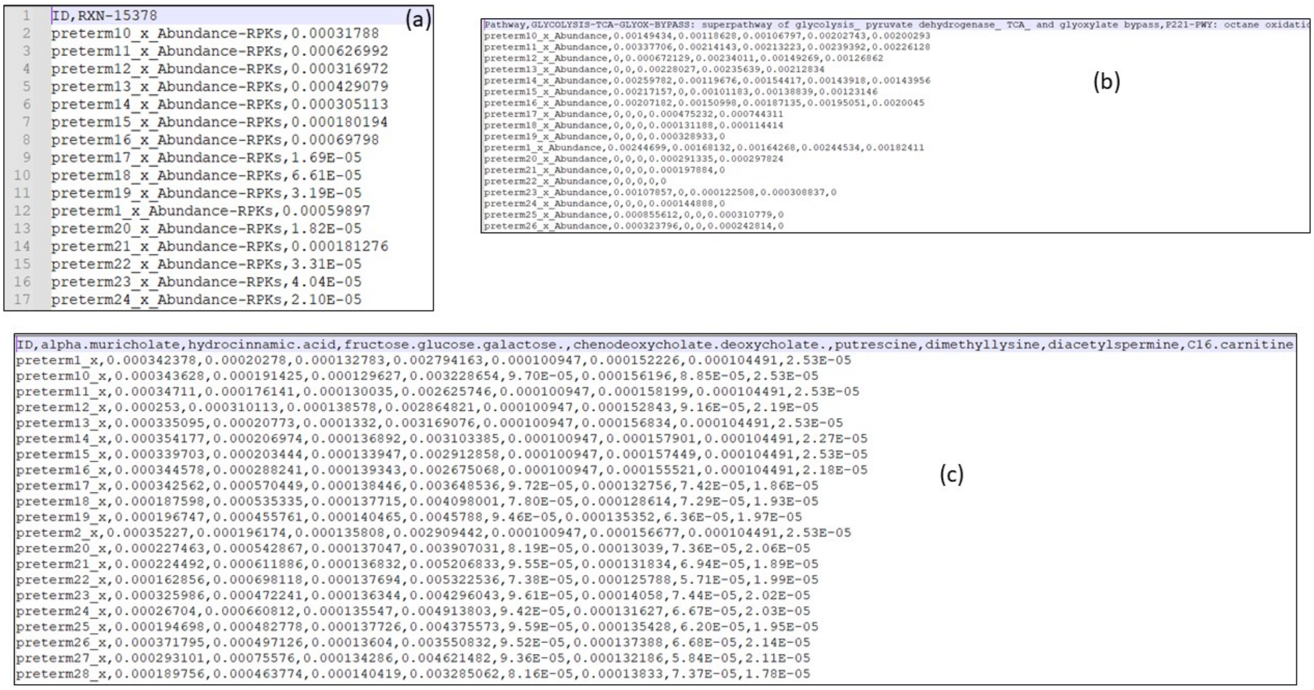

```
ID,RXN-15378
preterm10_x_Abundance-RPKs,0.00031788
preterm11_x_Abundance-RPKs,0.000626992
preterm12_x_Abundance-RPKs,0.000316972
preterm13_x_Abundance-RPKs,0.000429079
preterm14_x_Abundance-RPKs,0.000305113
preterm15_x_Abundance-RPKs,0.000180194
preterm16_x_Abundance-RPKs,0.00069798
preterm17_x_Abundance-RPKs,1.69E-05
preterm18_x_Abundance-RPKs,6.61E-05
preterm19_x_Abundance-RPKs,3.19E-05
preterm1_x_Abundance-RPKs,0.00059897
preterm20_x_Abundance-RPKs,1.82E-05
preterm21_x_Abundance-RPKs,0.000181276
preterm22_x_Abundance-RPKs,3.31E-05
preterm23_x_Abundance-RPKs,4.04E-05
preterm24_x_Abundance-RPKs,2.10E-05
```

(a)

```
Pathway,GLYCOLYSIS-TCA-GLYOX-BYPASS: superpathway of glycolysis_ pyruvate dehydrogenase_ TCA_ and glyoxylate bypass,P221-PWY: octane oxidati
preterm10_x_Abundance,0.00149434,0.00118628,0.00106797,0.00202743,0.00200293
preterm11_x_Abundance,0.00337706,0.00214143,0.00213223,0.00239392,0.00226128
preterm12_x_Abundance,0,0.000672129,0.00234011,0.00149269,0.00126862
preterm13_x_Abundance,0,0,0.00228027,0.00235639,0.00212834
preterm14_x_Abundance,0.00259782,0.00119676,0.00154417,0.00143918,0.00143956
preterm15_x_Abundance,0.00217157,0,0.00101183,0.00138839,0.00123146
preterm16_x_Abundance,0.00207182,0.00150998,0.00187135,0.00195051,0.0020045
preterm17_x_Abundance,0,0,0,0.000475232,0.000744311
preterm18_x_Abundance,0,0,0,0.000131188,0.000114414
preterm19_x_Abundance,0,0,0,0.000328933,0
preterm1_x_Abundance,0.00244699,0.00168132,0.00164268,0.00244534,0.00182411
preterm20_x_Abundance,0,0,0,0.000291335,0.000297824
preterm21_x_Abundance,0,0,0,0.000197884,0
preterm22_x_Abundance,0,0,0,0,0
preterm23_x_Abundance,0.00107857,0,0.000122508,0.000308837,0
preterm24_x_Abundance,0,0,0,0.000144888,0
preterm25_x_Abundance,0.000855612,0,0,0.000310779,0
preterm26_x_Abundance,0.000323796,0,0,0.000242814,0
```

(b)

```
ID,alpha.muricholate,hydrocinnamic.acid,fructose.glucose.galactose.,chenodeoxycholate.deoxycholate.,putrescine,dimethyllysine,diacetylspermine,C16.carnitine
preterm1_x,0.000342378,0.00020278,0.000132783,0.002794163,0.000100947,0.000152226,0.000104491,2.53E-05
preterm10_x,0.000343628,0.000191425,0.000129627,0.003228654,9.70E-05,0.000156196,8.85E-05,2.53E-05
preterm11_x,0.00034711,0.000176141,0.000130035,0.002625746,0.000100947,0.000158199,0.000104491,2.53E-05
preterm12_x,0.000253,0.000310113,0.000138578,0.002864821,0.000100947,0.000152843,9.16E-05,2.19E-05
preterm13_x,0.000335095,0.00020773,0.0001332,0.003169076,0.000100947,0.000156834,0.000104491,2.53E-05
preterm14_x,0.000354177,0.000206974,0.000136892,0.003103385,0.000100947,0.000157901,0.000104491,2.27E-05
preterm15_x,0.000339703,0.000203444,0.000133947,0.002912858,0.000100947,0.000157449,0.000104491,2.53E-05
preterm16_x,0.000344578,0.000288241,0.000139343,0.002675068,0.000100947,0.000155521,0.000104491,2.18E-05
preterm17_x,0.000342562,0.000570449,0.000138446,0.003648536,9.72E-05,0.000132756,7.42E-05,1.86E-05
preterm18_x,0.000187598,0.000535335,0.000137715,0.004098001,7.80E-05,0.000128614,7.29E-05,1.93E-05
preterm19_x,0.000196747,0.000455761,0.000140465,0.0045788,9.46E-05,0.000135352,6.36E-05,1.97E-05
preterm2_x,0.00035227,0.000196174,0.000135808,0.002909442,0.000100947,0.000156677,0.000104491,2.53E-05
preterm20_x,0.000227463,0.000542867,0.000137047,0.003907031,8.19E-05,0.00013039,7.36E-05,2.06E-05
preterm21_x,0.000224492,0.000611886,0.000136832,0.005206833,9.55E-05,0.000131834,6.94E-05,1.89E-05
preterm22_x,0.000162856,0.000698118,0.000137694,0.005322536,7.38E-05,0.000125788,5.71E-05,1.99E-05
preterm23_x,0.000325986,0.000472241,0.000136344,0.004296043,9.61E-05,0.00014058,7.44E-05,2.02E-05
preterm24_x,0.00026704,0.000660812,0.000135547,0.004913803,9.42E-05,0.000131627,6.67E-05,2.03E-05
preterm25_x,0.000194698,0.000482778,0.000137726,0.004375573,9.59E-05,0.000135428,6.20E-05,1.95E-05
preterm26_x,0.000371795,0.000497126,0.00013604,0.003550832,9.52E-05,0.000137388,6.68E-05,2.14E-05
preterm27_x,0.000293101,0.00075576,0.000134286,0.004621482,9.36E-05,0.000132186,5.84E-05,2.11E-05
preterm28_x,0.000189756,0.000463774,0.000140419,0.003285062,8.16E-05,0.00013833,7.37E-05,1.78E-05
```

(c)

**Figure 4.** Prepared datasets for enzymatic reactions (a), metabolic pathways (b) and predicted metabolites (c).

**Step 4**

After saving the dataset, the user must use the next command in the terminal:

```
java --enable-preview -jar jadbio-1.4.55-model-exe.jar -m <NAME OF THE MODEL>.bin -i <NAME OF PREPARED DATASET>.csv -o result.csv
```

This command runs the model on test data (<NAME OF PREPARED DATASET>.csv in our case) and creates a new dataset with predictions (result.csv) (Fig. 5).

```
C:\Users\LDeutsc\Desktop\preterm models>java --enable-preview -jar jadbio-1.4.55-model-exe.jar -m ecs.bin -i ECStest.csv -o ECSresults.csv
Successfully loaded model from ecs.bin
Successfully loaded input dataset from ECStest.csv
Successfully wrote predictions to ECSresults.csv
```
(a)

```
C:\Users\LDeutsc\Desktop\preterm models>java --enable-preview -jar jadbio-1.4.55-model-exe.jar -m pathways.bin -i Pathwaystest.csv -o Pathwaysresults.csv
Successfully loaded model from pathways.bin
Successfully loaded input dataset from Pathwaystest.csv
Successfully wrote predictions to Pathwaysresults.csv
```
(b)

```
C:\Users\LDeutsc\Desktop\preterm models>java --enable-preview -jar jadbio-1.4.55-model-exe.jar -m PredictedMetabolites.bin -i PredictedMetabolitestest.csv -o PredictedMetabolitesresults.csv
Successfully loaded model from PredictedMetabolites.bin
Successfully loaded input dataset from PredictedMetabolitestest.csv
Successfully wrote predictions to PredictedMetabolitesresults.csv
```
(c)

**Figure 5**. Executing the model and creating the result .csv file with predictions in the same directory based on a given enzymatic reactions (a), metabolic pathways (b) and predicted metabolites (c).

**Step 5**

After model execution, the user can check the calculated predictions by opening the .csv file directly by clicking on the created .csv file and opening it in any data analysis program (Excel, Past, R ...). As shown in Fig. 6 the model classifies the data between two groups (preterm and control). The first column is the same as in the test data created by the user.

(a)

| | A | B | C |
|---|---|---|---|
| 1 | Sample na | Prob ( class = Control ) | Prob ( class = Preterm ) |
| 2 | preterm10 | 0.332290189 | 0.667709811 |
| 3 | preterm11 | 0.022846072 | 0.977153928 |
| 4 | preterm12 | 0.33428622 | 0.66571378 |
| 5 | preterm13 | 0.14210351 | 0.85789649 |
| 6 | preterm14 | 0.360881065 | 0.639118935 |
| 7 | preterm15 | 0.660219252 | 0.339780748 |
| 8 | preterm16 | 0.0114512 | 0.9885488 |
| 9 | preterm17 | 0.907183957 | 0.092816043 |
| 10 | preterm18 | 0.857293973 | 0.142706027 |
| 11 | preterm19 | 0.893911389 | 0.106088611 |

(b)

| | A | B | C |
|---|---|---|---|
| 1 | Sample na | Prob ( class = Control ) | Prob ( class = Preterm ) |
| 2 | preterm10 | 0.439365634 | 0.560634366 |
| 3 | preterm11 | 0.311599427 | 0.688400573 |
| 4 | preterm12 | 0.510187055 | 0.489812945 |
| 5 | preterm13 | 0.481385161 | 0.518614839 |
| 6 | preterm14 | 0.437772542 | 0.562227458 |
| 7 | preterm15 | 0.521772789 | 0.478227211 |
| 8 | preterm16 | 0.394080668 | 0.605919332 |
| 9 | preterm17 | 0.64627694 | 0.35372306 |

(c)

| | A | B | C |
|---|---|---|---|
| 1 | Sample na | Prob ( class = Control ) | Prob ( class = Preterm ) |
| 2 | preterm1_ | 0.002657309 | 0.997342691 |
| 3 | preterm10 | 0.002889081 | 0.997110919 |
| 4 | preterm11 | 2.28E-05 | 0.999977227 |
| 5 | preterm12 | 0.958998991 | 0.041001009 |
| 6 | preterm13 | 9.09E-04 | 0.999090718 |
| 7 | preterm14 | 1.49E-04 | 0.999851037 |
| 8 | preterm15 | 2.34E-04 | 0.999765739 |
| 9 | preterm16 | 0.003400977 | 0.996599023 |
| 10 | preterm17 | 0.999999999 | 1.01E-09 |
| 11 | preterm18 | 1 | 1.85E-14 |
| 12 | preterm19 | 1 | 1.53E-12 |

**Figure 6.** The newly created .csv file with predictions calculated from enzymatic reactions (a), metabolic pathways (b) and predicted metabolites (c).
